# Design and methodology of a continuous national dietary and nutrition survey in France: the Nutrimetry study

**DOI:** 10.3389/fnut.2026.1909933

**Published:** 2026-08-24

**Authors:** Elena Sauvage, Jean-Michel Lecerf, Victoria Dupuis, Coralie Berthier, Pascale Hébel

**Affiliations:** 1 Trend & People, C-ways, Paris, France; 2 Nutrition Department, Institut Pasteur de Lille, Lille, France

**Keywords:** diet surveys, energy intake, feeding behaviour, methodology, nutrition surveys

## Abstract

**Objective:**

Assessing dietary intake and nutritional status requires contemporary data. This paper describes the design of the Nutrimetry survey, a new continuous monitoring system in France, its participation rate, and the quality of its dietary data.

**Design:**

A representative sample of adults (18–79 years) and children (3–17 years) was selected using a non-probability quota sampling design. Quotas included age, sex, region, urban unit size, and socio-professional category, weighted against 2021 census data.

**Setting:**

Food consumption was collected via a 3-day online dietary record. Information on physical activity, sedentary behaviour, and sociodemographic was gathered by questionnaires. Complex foods were disaggregated into raw ingredients using a standardized recipe optimization process.

**Participants:**

In total, 3,201 individuals (2,193 adults and 1,008 children) were included in 2024 results.

**Results:**

The final sample represented 7% of adult starters and 45.2% of eligible children. Mean energy intake was 1,852 kcal/d in adults and 1,693 kcal/d in children. The prevalence of energy under-reporting was 33.8% for adults and 22.6% for children. Under-reporting was significantly associated with higher BMI and younger age groups.

**Conclusion:**

The Nutrimetry survey provides a robust, scalable framework for continuous national dietary surveillance in France. Despite challenges with online recruitment attrition, it generates timely data for public health policy and nutritional risk assessment.

## 1 Introduction

The assessment of dietary intake and nutritional status represents a major public health priority. It enables the evaluation of diet-related risks and benefits, supports the development of evidence-based nutritional recommendations, and informs prevention policies. The reliability of such evaluations depends on the availability of accurate and representative data.

In France, several national surveys have documented these dimensions. The Individual and National Food Consumption Surveys (INCA) (1–3), conducted by ANSES between 1998 and 2015 (INCA1–3), provided detailed information on food consumption, nutrient intakes, and the nutritional status of the French population. The CCAF surveys (Comportements et Consommations Alimentaires des Français) (4, 5), conducted between 2003 and 2019, also provided complementary data—particularly until 2013—on cooking practices, eating behaviours, and consumption habits. Together, these surveys revealed substantial shifts in dietary habits and behaviours over time, highlighting the need for regular data updates to maintain a comprehensive and contemporary overview of national nutritional trends.

Internationally, several nutrition surveillance systems routinely generate reference data. The United Kingdom’s National Diet and Nutrition Survey (NDNS) (6) provides continuous monitoring of dietary intake and nutritional status, enabling meaningful international comparisons. At the European level, the European Food Safety Authority (EFSA) launched the EU Menu project to harmonize food consumption data collection across Member States (7, 8). Beyond Europe, Mexico’s National Health and Nutrition Survey (Encuesta Nacional de Salud y Nutrición, ENSANUT), coordinated by the National Institute of Public Health, provides a further reference for continuous nutritional surveillance: since 2020, ENSANUT has moved from a periodic (six-year) design to a continuous, annually collected model (Ensanut Continua 2020–2024) (9). A distinctive feature of ENSANUT is that it complements self-reported dietary intake with objective measurements—including measured anthropometry and biological sampling for indicators such as lipid profiles and, in children, blood lead concentration—thereby linking food consumption to clinical and environmental markers of nutritional status (9, 10). Together, these initiatives underscore the importance of regular updates, methodological standardization, and, where feasible, the integration of biological measurements to ensure data comparability and quality.

In this context, the Nutrimetry survey was designed to generate contemporary, continuous, and representative data on the French population concerning foods and beverages consumed; nutrient intakes and their adequacy to recommendations; indicators of nutritional status, including body mass index (BMI); and physical activity and sedentary behaviours. The present article delineates the Nutrimetry survey methodology, examines participation rates, and provides a first assessment of the quality of the dietary data collected.

## 2 Materials and methods

### 2.1 Study design

Nutrimetry is a cross-sectional, continuous survey conducted throughout mainland France since February 2024. Its objective is to provide ongoing monitoring of dietary intake and related health indicators in the French population. The present article describes the methodology and first quality indicators based on data collected in 2024.

### 2.2 Testing stage

A pilot study was conducted in January 2024 among 756 individuals to determine the appropriate incentive level and assess completion and dropout rates. The overall completion rate was 45% (38% among adults and 61% among children). Chi-squared tests showed no significant effect of incentive amount (€20 vs. €40) on completion rates (adults: 35% vs. 43%, *p* = 0.073; children: 60% vs. 64%, *p* = 0.501). However, not imposing specific diary days significantly improved completion among adults (33% with imposed days vs. 45% with flexible days, *p* = 0.005), while the effect was not statistically significant among children (56% vs. 67%, *p* = 0.100). Based on these findings, the incentive for the main study was set at €20 and no specific recording days were imposed.

### 2.3 Sampling design

The study used a non-probability quota sampling design. The target population included children aged 3–17 years and adults aged 18–79 years residing in mainland France. For children, quotas were defined according to household type, urban unit size, region, socio-professional category of the household head (INSEE classification) (11), and age stratified by sex. For adults, quotas were established based on socio-professional category (including former occupation), type of household, age, sex, region, and urban unit size, in order to reflect the socio-demographic structure of the French population. Reference distributions were derived from the 2021 INSEE census (11). To account for seasonal variation, recruitment was balanced across quarters. The study aimed to enrol 250 children and 500 adults per quarter, resulting in a target sample of 3,000 individuals for 2024. This sample size met EFSA minimum requirements for estimating high percentiles within age- and sex-specific subgroups (7).

### 2.4 Recruitment

Participants were recruited online through targeted advertisements on Facebook and Instagram, stratified by age group. Eligibility criteria were intentionally broad: participants had to reside in mainland France and to be within the target age ranges (adults aged 18–79 years and children aged 3–17 years), and the respondent—or, for children, the parent or legal guardian completing the survey—had to be able to read and complete the questionnaire in French. No further eligibility restrictions were applied; in particular, there was no exclusion based on pregnancy or lactation and no minimum duration-of-residency requirement. This social media–based recruitment strategy was selected for its capacity to reach large, diverse, and otherwise hard-to-reach segments of the population across all geographic areas and socio-demographic groups, without relaxing the eligibility or targeting criteria. Compared with recruitment relying on a single pre-recruited access panel—which tends to over-represent particular demographic or behavioural profiles and may thereby introduce selection bias—quota-based targeted advertising across social networks broadens the pool of potential respondents and limits single-source bias (12). When combined with the quota framework described above and the post-stratification weighting detailed below, this approach enables the constitution of samples that closely reflect the socio-demographic structure of the target population while supporting rapid and continuous data collection. Social media–based recruitment has been increasingly applied in large-scale health and population surveys, where, combined with targeted quotas, appropriate quality controls, and post-stratification weighting, it can yield samples of representativeness comparable to that obtained with more traditional recruitment approaches (13). All participants provided informed consent prior to participation. For children aged 3–17 years, a parent or legal guardian completed the questionnaires and dietary records on behalf of the child. To avoid clustering effects, only one participant per household was included.

### 2.5 Data collection and measurements

Participants first completed a self-administered online recruitment questionnaire collecting socio-demographic information required to establish quotas and verify eligibility. Upon completion, participants accessed a secure online platform to complete a three-day food diary. After completing the dietary record, participants filled in a final questionnaire covering eating habits and lifestyle characteristics. Use of dietary supplements (vitamins or minerals) was not recorded, and no clinical information—such as chronic conditions (e.g., diabetes, coeliac disease) or appetite- or metabolism-altering medication—was collected; these variables were therefore not available for the nutritional assessment. All collected variables are summarised in Table 1.

**Table 1:** Summary of data collected in the Nutrimetry survey and corresponding data collection methods.

Type of data collected	Description of the collected data	Data collection method
Food consumption	All foods and beverages recorded over 3 days; quantities, brands, labels and product characteristics; occasions and places of consumption; context of consumption (seated or standing, use of cutlery, eating companions, meal duration, screen use)	Online food diary
Socio-demographic context	Socio-demographic characteristics of the participant and head of household	Recruitment questionnaire
Physical activity and sedentary behaviour	Physical activity (domestic, occupational and leisure activities); sedentary behaviour (screen time, sedentary time at work, during leisure activities and transportation)	End-of-study questionnaire
Anthropometric characteristics and health status	Body weight and height; smoking status; sleep; women-specific information (menopause, pregnancy, breastfeeding, menstrual flow)	End-of-study questionnaire
Additional information potentially affecting diet	Fat consumption habits; adherence to a special diet; food insecurity; economic hardship; use of meal vouchers (“restaurant vouchers”); presence of a home garden	End-of-study questionnaire

#### 2.5.1 Dietary data collection

Detailed information on food and beverage consumption was collected over 3 days using an online dietary record adapted from the INCA (14) and CCAF (5, 15) surveys. A three-day recording period was selected to reduce respondent burden and limit under-reporting commonly observed with seven-day records (16). Each recording day was divided into 10 eating occasions: three main meals (breakfast, lunch, and dinner) and seven potential snack occasions (including aperitifs). Standardised portion photographs from the SUVIMAX study were provided to assist portion size estimation (17). Participants directly entered information on food items, quantities, brands, and contextual characteristics into the online diary. To minimise implausible reporting, a minimum energy intake threshold of 750 kcal/day was applied. During completion of the food diary, participants were excluded if their reported intake fell below this threshold on at least two of the three recorded days. The participant-facing wording in the recruitment questionnaire refers to this same 750 kcal/day rule as a condition of eligibility for the participation incentive; it is not a separate criterion. Additionally, the method proposed by Yang et al. (18) was applied after data collection as an additional plausibility filter: participants whose mean daily energy intake across the three recording days was below 500 kcal/day were excluded (n = 3).

#### 2.5.2 Anthropometric, and lifestyle indicators

Participants self-reported their height and weight, from which BMI was calculated according to WHO criteria (19): underweight (<18.5 kg/m^2^), normal weight (18.5–24.9 kg/m^2^), overweight (25–29.9 kg/m^2^), and obesity (≥30 kg/m^2^). Physical activity and sedentary behaviour were assessed using the International Physical Activity Questionnaire (IPAQ) (20). Participants also reported screen time and average sleep duration. Based on National Sleep Foundation recommendations (21), sleep duration was categorised into two groups: <7 h per night and ≥7 h per night.

#### 2.5.3 Eating habits

Fat consumption habits were assessed using frequency-based questions on high-fat foods and cooking practices. Adherence to medically prescribed or self-selected special diets was recorded. Food insecurity was measured using the validated French version of the U. S. Household Food Security Survey Module (22), six-item short form. Participants also reported financial hardship, use of subsidised meal vouchers (“ticket restaurant”), and the presence of a home garden.

### 2.6 Data analysis

#### 2.6.1 Weighting

Post-stratification is a simple and robust method for improving the representativeness of a survey sample, particularly in epidemiology where certain sub-populations are often under- or over-represented. It adjusts the weights of individuals so that the distribution of the sample corresponds to that of the population on key variables (e.g., age, gender, region). This technique reduces estimation biases related to imperfect coverage or non-response, while preserving the structure of the sampling plan. It is particularly useful when the adjustment variables are strongly correlated with the variable of interest (prevalence, exposure, etc.), which improves the external validity of the results. According to Korn and Graubard (23), post-stratification can significantly reduce biases due to differential non-response and provide more accurate estimates of population parameters. Thanks to this process, the final samples were representative for the variables used for the calibration at the national level. Both final individual weighting factors and the sampling design effect were considered for appropriate individual data analysis.

#### 2.6.2 Assessment of food and nutrient intakes

To ensure comparability with the INCA3 survey, all reported foods and beverages were coded according to the EFSA FoodEx2 classification system (7). Nutrient intakes were calculated using a food composition database developed specifically for Nutrimetry, based on the 2020 CIQUAL database (24), complemented by CALNUT 2020 data (25) and manufacturer information. The database included 54 nutrients, covering energy, macronutrients, 11 minerals, 13 vitamins, and 14 fatty acids.

#### 2.6.3 Recipe decomposition

**Figure 1:**
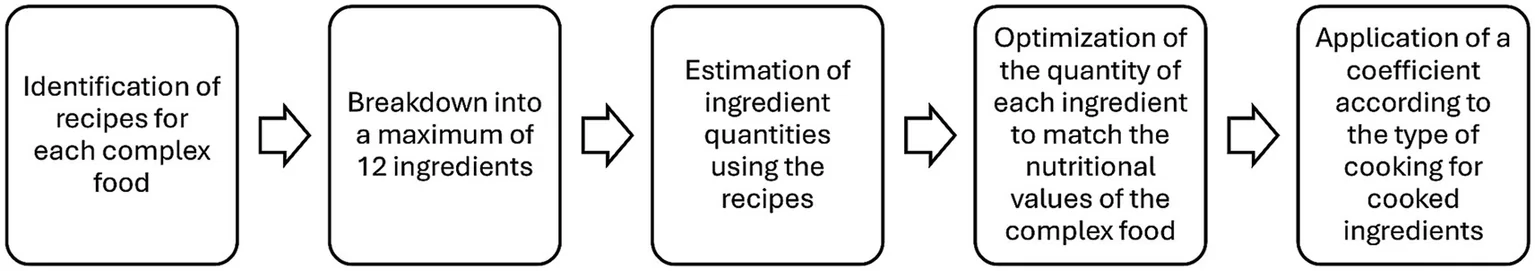
Overview of the Nutrimetry survey design and data collection framework.

Complex foods were disaggregated into raw ingredients to allow detailed analysis of food group consumption. Recipes for industrial products were reconstructed using product information from specialised databases and online retailers, while homemade dishes were based on widely consulted culinary sources. Nutritional optimisation was performed using seven key nutrients (energy, water, protein, carbohydrates, fibre, calcium, and vitamin B12) to ensure consistency with CIQUAL reference values (24). Cooking yield factors were applied based on published data (26, 27). This processing step forms part of the overall survey design and data-collection framework summarised in Figure 1.

#### 2.6.4 Quality indicators for dietary data

At the individual level, dietary data quality was assessed using mean energy intake (MEI), the ratio of MEI to estimated basal metabolic rate (MEI: BMR), and the prevalence of under-reporting. BMR was estimated using the age- and sex-specific Henry equations (28). Physical activity level (PAL) was derived from the International Physical Activity Questionnaire (IPAQ) (20) following the standard IPAQ scoring protocol: for each adult, an IPAQ score (in MET-minutes/week) was computed as 8.0 × vigorous activity (min/week) + 4.0 × moderate activity (min/week) + 3.3 × walking (min/week), and participants were classified as low/inactive (score < 600), moderate/active (score 600–2,999), or high/very active (score ≥ 3,000). Each category was assigned a PAL constant of 1.40 (low), 1.60 (moderate), or 1.80 (high). Under-reporting was identified using the Goldberg cut-off method, as recommended by EFSA (29–31), by comparing each individual’s MEI: BMR ratio with the PAL-specific ±2 SD confidence limits of Black (32). The lower and upper limits were 0.91 and 2.16 for a PAL of 1.40, 1.04 and 2.47 for a PAL of 1.60, and 1.16 and 2.78 for a PAL of 1.80. Participants were classified as under-reporters when their MEI: BMR fell below the lower limit, as plausible reporters when it lay between the two limits, and as over-reporters when it exceeded the upper limit; only under-reporters were excluded from subsequent analyses. Statistical analyses were performed using Python (statsmodels package). A two-sided *p*-value < 0.05 was considered statistically significant.

## 3 Results

### 3.1 Participation rate

**Figure 2:**
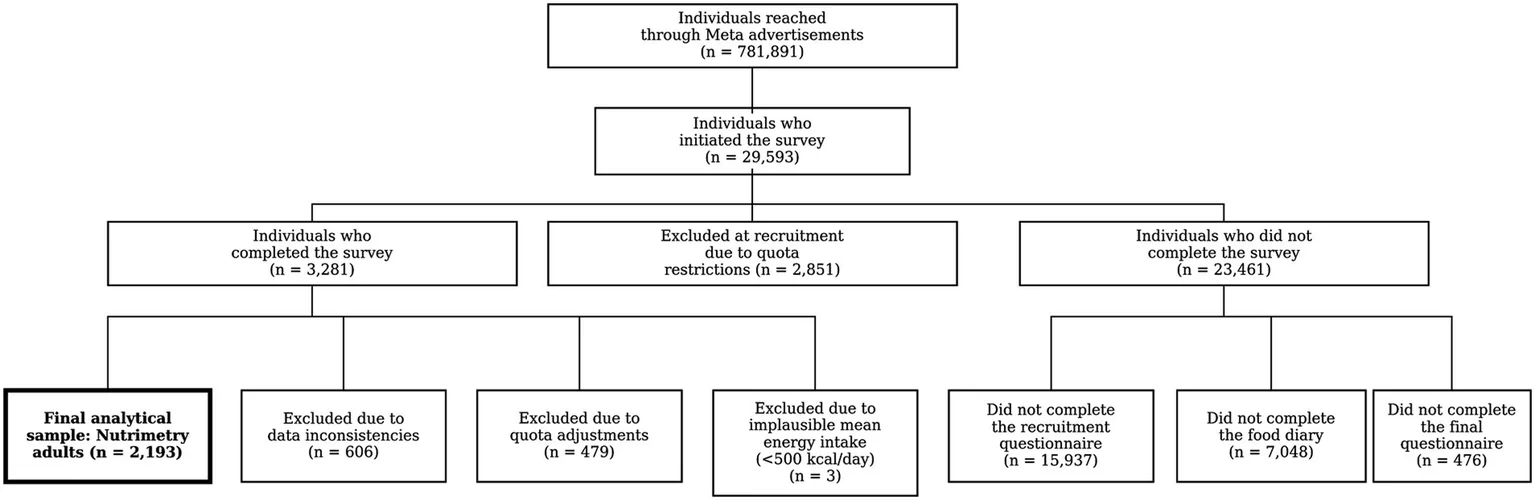
Participant flow diagram for the Nutrimetry survey (2024), showing recruitment, attrition, and final sample sizes for the adult and child samples.

Figure 2 illustrates the participant flow. Recruitment for the adult sample began with a large-scale advertising campaign on Meta platforms, reaching 781,891 individuals. Of these, 4% (n = 29,593) initiated the survey and 11% of starters (n = 3,281) completed it. Most of the attrition occurred among participants who began but did not complete the survey (79%), mainly due to incomplete recruitment questionnaires (68%) or incomplete food diaries (30%). Three participants were excluded at the plausibility-check stage because their mean daily energy intake across the three recording days was below 500 kcal/day. After applying eligibility criteria and quota adjustments, the final adult sample comprised 2,193 participants. Exclusions were due to data inconsistencies (19%) and additional quota adjustments (15%). Overall, the final sample represented 7% of individuals who initially started the survey. For the child sample, 2,228 parents completed the recruitment questionnaire. Among them, 762 children (34.2%) were excluded due to quota restrictions. A total of 1,008 children were ultimately included in the survey. An additional 458 eligible children were lost due to non-completion of the food diary or final questionnaire. The final child sample therefore represented 45.2% of parents who completed the recruitment questionnaire.

### 3.2 Description of participants

The unweighted socio-demographic characteristics of the child and adult samples are presented in Tables 2, 3, respectively, and compared with national population data.

**Table 2:** Raw distribution (%) of participants in the Nutrimetry child sample by socio-demographic characteristics, compared with national population statistics (mainland France).

Socio-demographic characteristic	Nutrimetry 2024	Population of mainland France
	(*n* = 1,008)	
Sex and age group		
Girls	50.0%	48.9%
3–5 years old	11.7%	9.0%
6–8 years old	10.8%	9.6%
9–14 years old	18.8%	20.2%
15–17 years old	8.6%	10.1%
Boys	50.0%	51.1%
3–5 years old	11.0%	9.6%
6–8 years old	10.4%	10.0%
9–14 years old	19.4%	21.2%
15–17 years old	9.1%	10.3%
Agglomeration size		
Rural municipality	18.5%	22.3%
Municipality with fewer than 20,000 inhabitants	37.3%	18.4%
Municipality with 20,000 to 100,000 inhabitants	15.9%	13.2%
Urban area with more than 100,000 inhabitants	16.1%	28.9%
Parisian urban area	12.3%	17.3%
Type of household		
Single (and others)	0.1%	2.5%
Couple with child(ren)	83.5%	76.6%
Single parent	16.4%	20.9%
Socio-professional class of the head of the household		
Craftsmen, Tradesmen, Business Managers and Farmers	6.0%	10.4%
Executives and Senior Professionals	14.0%	19.1%
Intermediate Professionals	14.9%	22.1%
Employees	43.8%	17.1%
Labourers	14.1%	29.2%
Inactive	7.2%	2.2%
Area		
Eastern Paris Basin	9.0%	7.5%
Western Paris Basin	10.7%	9.1%
East	8.6%	8.1%
Mediterranean	11.0%	11.9%
North	14.0%	6.8%
West	10.8%	13.9%
Paris Region	14.8%	19.8%
South-East	12.4%	12.6%
South-West	8.6%	10.3%

**Table 3:** Raw distribution (%) of participants in the Nutrimetry adult sample by socio-demographic characteristics, compared with national population statistics (mainland France).

Socio-demographic characteristic	Nutrimetry 2024	Population of mainland France
	(*n* = 2,194)	
Gender		
Men	37.42%	48.75%
Women	62.58%	51.25%
Age		
18–34 years old	26.8%	27.0%
35–49 years old	33.5%	25.9%
50–64 years old	25.1%	26.5%
65–79 years old	14.6%	20.6%
Agglomeration size		
Rural municipality	18.4%	21.2%
Municipality with fewer than 20.000 inhabitants	30.7%	17.8%
Municipality with 20.000 to 100.000 inhabitants	14.9%	13.5%
Urban area with more than 100.000 inhabitants	20.9%	30.5%
Parisian urban area	15.1%	16.9%
Type of household		
Single	22.3%	19.9%
Couple without children	30.6%	28.2%
Couple with child(ren)	32.0%	37.3%
Single with child(ren)	10.5%	9.7%
Others	4.6%	4.9%
Socio-professional class		
Craftsmen, Tradesmen, Business Managers and Farmers	4.8%	7.4%
Executives and Senior Professionals	16.7%	15.3%
Intermediate Professionals	16.3%	21.8%
Employees	38.6%	26.1%
Labourers	8.6%	20.2%
Inactive	15.0%	9.2%
Area		
Eastern Paris Basin	7.9%	7.4%
Western Paris Basin	8.8%	8.9%
East	7.5%	8.4%
Mediterranean	11.9%	12.7%
North	8.7%	6.2%
West	12.1%	13.7%
Paris Region	17.3%	19.1%
South-East	15.0%	12.3%
South-West	10.8%	11.3%

In the child sample, the sex distribution closely reflected national statistics (50.0% girls, 50.0% boys). Younger children (3–8 years) were slightly overrepresented, whereas adolescents (9–17 years) were somewhat underrepresented. Smaller municipalities and two-parent households were overrepresented, while large urban areas and single-parent households were underrepresented. Employees were overrepresented relative to national distributions, whereas executives, intermediate professionals, and labourers were underrepresented. Regionally, the Paris Region was slightly underrepresented and the North region overrepresented.

In the adult sample, women were overrepresented (62.6% vs. 51.3% nationally), while men were underrepresented. Younger adults (18–34 years) were proportionally represented, individuals aged 35–49 years were slightly overrepresented, and those aged 65–79 years were underrepresented. Residents of municipalities with fewer than 20,000 inhabitants were overrepresented, whereas those living in large urban areas were underrepresented. Employees and executives were overrepresented, while labourers were markedly underrepresented. After application of post-stratification weights, the sample distribution aligned with national population benchmarks.

### 3.3 Dietary data quality

The three-day food diary protocol yielded 3,024 diary days for children and 6,579 diary days for adults (Table 4). Recordings were distributed across all days of the week, capturing both routine and variable consumption patterns. Among adults, diary days were slightly more concentrated toward the end of the week (Thursday–Sunday), whereas among children, recordings were more frequent at the beginning of the week (Monday–Tuesday), reflecting school-related routines. Seasonally, spring accounted for approximately 37% of diary days in both groups. Combined, spring, summer, and autumn represented approximately 87% of recordings. Winter was underrepresented, as January was devoted to the pilot phase. Nevertheless, the broad seasonal coverage supports the estimation of annual dietary intake. Mean energy intake (EI) was 1,693 kcal/day in children (Table 5) and 1,852 kcal/day in adults (Table 6). As expected, EI was consistently lower in females than in males, increased with age throughout childhood and adolescence, and declined in older adults. The mean EI: BMR ratio was 1.41 in children and 1.22 in adults. Among adults, EI: BMR was slightly higher in men than in women in younger age groups but showed no overall sex difference. In children, EI: BMR increased with age and was slightly higher in girls than in boys overall. The prevalence of under-reporting was 22.6% in children and 33.8% in adults. In children, under-reporting increased markedly with age, rising from 17.6% among those aged 3–5 years to 36.8% among adolescents aged 15–17 years, reaching 40.0% in adolescent boys. In adults, under-reporting ranged from 31.0 to 36.8% across age groups, with slightly higher rates among men overall. Two subgroups exhibited particularly high levels of under-reporting: men aged 35–49 years (42.1%) and boys aged 15–17 years (40.0%). These findings highlight substantial demographic disparities in reporting accuracy and underscore the importance of accounting for misreporting when interpreting dietary intake data.

**Table 4:** Description of food diary recordings in the Nutrimetry survey by study sample.

Characteristic	Children (*n* = 3,024 diary days)	Adults (*n* = 6,579 diary days)
Distribution by day%		
Monday	16.9	13.6
Tuesday	16.1	13.7
Wednesday	14.9	13.9
Thursday	11.3	14.7
Friday	12.1	15.7
Saturday	13.6	14.2
Sunday	15.2	14.2
Distribution by season%		
Winter	12.8	6.5
Spring	37.4	37.5
Summer	26.7	29.3
Autumn	23	26.7

Values are percentages; *n* corresponds to the number of recorded diary days, not the number of participants.

**Table 5:** Energy intake and prevalence of energy under-reporting among children aged 3–17 years in the Nutrimetry survey (*n* = 1,008).

Outcome	Age group (years)	Boys	Girls	Total	*p*-value
Energy intake (kcal/d), mean (SD)	3–5	1,304 (329)	1,451 (520)	1,376 (439)	*
	6–8	1718 (408)	1,486 (377)	1,605 (410)	***
	9–14	1775 (531)	1754 (494)	1764 (513)	NS
	15–17	2042 (1022)	1773 (629)	1911 (864)	*
	All	1734 (664)	1,649 (529)	1,693 (603)	*
EI: BMR, mean (95% CI)	3–5	1.43 (1.33–1.54)	1.62 (1.49–1.75)	1.53 (1.44–1.61)	***
	6–8	1.56 (1.38–1.74)	1.46 (1.38–1.55)	1.51 (1.41–1.61)	NS
	9–14	1.33 (1.24–1.41)	1.43 (1.35–1.51)	1.38 (1.32–1.44)	*
	15–17	1.20 (1.04–1.37)	1.30 (1.15–1.44)	1.25 (1.14–1.36)	NS
	All	1.37 (1.30–1.43)	1.45 (1.39–1.50)	1.41 (1.36–1.45)	***
Under-reporting (%), 95% CI	3–5	20.9 (10.0–31.8)	14.2 (5.7–22.7)	17.6 (10.7–24.5)	NS
	6–8	15.0 (0.0–31.0)	12.8 (4.6–21.1)	14.0 (5.0–22.9)	NS
	9–14	30.7 (21.2–40.3)	12.3 (5.9–18.8)	21.7 (15.9–27.6)	***
	15–17	40.0 (26.3–53.6)	33.4 (18.6–48.1)	36.8 (26.7–46.9)	NS
	All	27.8 (21.3–34.2)	17.1 (12.5–21.7)	22.6 (18.6–26.5)	***

Values are means (SD), means (95% CI) or percentages (95% CI), as indicated. *p*-value for sex differences within age groups (*p < 0.05; ***p < 0.001; NS, not significant). EI, energy intake; BMR, basal metabolic rate. To convert kcal to kJ, multiply by 4.184.

**Table 6:** Energy intake and prevalence of energy under-reporting among adults aged 18–79 years in the Nutrimetry survey (*n* = 2,194).

Outcome	Age group (years)	Men	Women	Total	*p*-value^†^
Energy intake (kcal/d), mean (SD)					
	18–34	2,209 (962)	1714 (589)	1957 (832)	***
	35–49	2001 (655)	1,699 (586)	1835 (636)	***
	50–64	2056 (620)	1,591 (570)	1823 (639)	***
	65–79	1940 (582)	1,601 (488)	1776 (565)	***
	All	2059 (736)	1,656 (567)	1852 (686)	***
EI: BMR, mean (95% CI)					
	18–34	1.31 (1.17–1.45)	1.23 (1.15–1.30)	1.26 (1.19–1.34)	NS
	35–49	1.16 (1.10–1.23)	1.24 (1.18–1.30)	1.21 (1.16–1.25)	***
	50–64	1.20 (1.13–1.28)	1.17 (1.08–1.26)	1.19 (1.13–1.24)	NS
	65–79	1.19 (1.10–1.28)	1.27 (1.19–1.34)	1.23 (1.17–1.29)	NS
	All	1.22 (1.17–1.27)	1.22 (1.19–1.26)	1.22 (1.19–1.25)	NS
Under-reporting (%), 95% CI					
	18–34	33.2 (22.9–43.4)	40.4 (31.9–48.8)	36.8 (30.2–43.5)	***
	35–49	42.1 (33.9–50.2)	29.2 (23.2–35.2)	35.0 (30.0–39.9)	***
	50–64	29.7 (21.1–38.3)	33.5 (23.3–43.8)	31.6 (24.9–38.3)	NS
	65–79	36.5 (24.8–48.2)	25.2 (16.5–33.9)	31.0 (23.7–38.4)	***
	All	35.1 (30.2–39.9)	32.5 (28.3–36.8)	33.8 (30.5–37.0)	NS

Values are means (SD), means (95% CI) or percentages (95% CI), as indicated. ^†^*p*-value for sex differences within age groups (***p < 0.001; NS, not significant). EI, energy intake; BMR, basal metabolic rate. To convert kcal to kJ, multiply by 4.184.

### 3.4 Analysis of under-reporters

Table 7 presents the results of a logistic regression analysis examining associations between under-reporting, age group, and BMI. Compared with adults aged 18–34 years (reference group), older adults (50–64 and 65–79 years) had significantly lower odds of under-reporting. A strong positive gradient was observed for BMI. Overweight and obese individuals were significantly more likely to under-report energy intake, whereas underweight individuals were less likely to do so. Overall, the probability of under-reporting increased with higher BMI and decreased with age. In terms of dietary differences, plausible reporters consumed substantially greater amounts of snack-type foods compared with under-reporters. For example, mean ice cream consumption was 3.6 g/day among plausible reporters versus 1.6 g/day among under-reporters. Similarly, confectionery and chocolate intake averaged 4.0 g/day versus 2.3 g/day, and pastry consumption averaged 60.3 g/day versus 35.2 g/day. These findings suggest selective under-reporting of energy-dense snack foods.

**Table 7:** Odds ratios and *p*-values for under-reporting status according to age group and body mass index (BMI) category (logistic regression).

Predictor	Odds ratio	*p*-value
Age group		
18–34 years (reference)	1.00	—
35–49 years	0.87	0.28
50–64 years	0.43	0.009
65–79 years	0.69	0.008
Body mass index		
Normal weight (reference)	1.00	—
Underweight	0.53	0.026
Overweight	1.28	0.024
Obesity	1.86	<0.001

Model: under-reporter = α + β₁ × age group + β₂ × BMI category. An odds ratio (OR) > 1 indicates a higher probability of under-reporting relative to the reference category; OR < 1 indicates a lower probability (e.g., OR = 1.86 for obesity ≈ 86% higher odds than normal weight). Reference categories: 18–34 years and normal weight. *p*-values < 0.05 indicate statistical significance.

## 4 Discussion

### 4.1 Participation and sampling

The Nutrimetry survey demonstrates the feasibility of continuous, web-based national dietary monitoring in France. However, participant retention remains a key challenge, particularly among adults, as only approximately 7% of initial starters were included in the final sample. Attrition was primarily attributable to incomplete recruitment questionnaires and food diaries. To better understand and ultimately reduce this attrition, future applications of the instrument would benefit from a structured follow-up of non-completers—for example, a short exit questionnaire triggered at the point of drop-off to capture the reasons for non-completion, such as limited comprehension of a specific question, perceived respondent burden, time constraints, or technical difficulties. Such insight would help identify which stages of the questionnaire and diary drive drop-out and would support iterative refinement of the administration format and question wording.

Before weighting, the adult sample included a higher proportion of women, residents of smaller municipalities, and employees, while men, older adults, and residents of large urban areas were underrepresented. These patterns likely reflect differences in engagement with online recruitment platforms and interest in participating in detailed dietary surveys. Although post-stratification weighting corrected major socio-demographic imbalances, residual bias related to unmeasured characteristics—such as health consciousness or dietary behaviours—cannot be excluded. Similar challenges have been reported in other national dietary surveys, including the UK National Diet and Nutrition Survey (NDNS) (33), where non-response bias is also a recognised limitation despite mixed-mode recruitment strategies.

Recruitment through social-media platforms (Facebook and Instagram) was effective for reaching large and diverse segments of the population, but it introduces a coverage bias that post-stratification weighting cannot fully remove. Weighting can align the number of participants in each age band with 2021 INSEE figures, but it cannot correct for the ways in which recruited individuals differ from their non-participating peers. This is particularly relevant for the oldest participants: individuals aged 60–79 years who respond to a social-media advertisement and complete a multi-day online diary are likely to be more digitally literate, and probably more health-conscious, than the average person in that age band. Estimates for the oldest groups should therefore be interpreted with caution. In addition, recruitment did not include platforms such as TikTok or Snapchat, which are more heavily used by adolescents; this may under-represent certain younger profiles. Diversifying recruitment channels across multiple platforms could, in principle, help improve coverage across age groups, although this is beyond the scope of the current design.

### 4.2 Comparison with existing dietary surveillance systems

Nutrimetry shares the continuous, regularly updated philosophy of leading international surveillance systems while differing from them in the range of variables collected. Compared with the French INCA surveys and with international instruments such as the NDNS, the EFSA EU Menu programme, and Mexico’s ENSANUT, Nutrimetry captures dietary intake together with age, sex, and several socio-economic dimensions (socio-professional category, household type, urban unit size, food insecurity assessed with the validated Household Food Security Survey Module, financial hardship, meal-voucher use, and the presence of a home garden). By contrast, it does not collect psycho-emotional variables (e.g., eating-related stress, emotional eating, or validated psychometric scales), nor clinical or biological markers. This contrasts most clearly with ENSANUT, which since 2020 has operated as a continuous, annually collected survey (Ensanut Continua 2020–2024) and complements self-reported intake with measured anthropometry and biological sampling, including lipid profiles and blood lead concentration in children (9, 10). More broadly, the integration of psycho-emotional measures or of objective clinical or biological components would enrich the information provided by this type of instrument, while acknowledging that such additions entail substantially greater participant burden and cost and are not part of the present design.

### 4.3 Dietary quality

The three-day dietary record provided broad temporal and seasonal coverage, although winter was underrepresented during the first year owing to the pilot phase conducted in January. As data accumulate across the continuous collection, seasonal coverage is expected to become more balanced. Under-reporting of energy intake is a well-documented limitation of self-reported dietary assessment methods. The prevalence observed in adults (33.8%) is consistent with previous literature, where approximately one-third of adults are typically classified as low-energy reporters (33, 34). Importantly, under-reporting is not random: it was more frequent among individuals with higher BMI and among specific demographic subgroups, and it may disproportionately affect energy-dense or socially undesirable foods. To make fuller use of these data and to provide a benchmark for other European studies, we now report the distribution of under-, plausible-, and over-reporters cross-classified by sex, age band, and BMI category (clinical cut-offs for adults; WHO/IOTF percentiles for children and adolescents) in Table 8. The observed pattern of lower reported intake of snack foods among under-reporters is consistent with the selective misreporting described in previous validation studies. As in other national surveys, under-reporters were retained in the descriptive analyses to preserve representativeness; however, their presence should be considered when interpreting absolute intake estimates.

**Table 8:** Distribution of under-, plausible-, and over-reporters by sex, age group, and BMI category, in adults and in children and adolescents.

Category	*n*	Under-reporters	Plausible reporters	Over-reporters
Adults (18–79 years)				
By sex				
Female	1,373	32.0%	66.4%	1.6%
Male	821	35.8%	61.8%	2.4%
By age group				
18–34 years	589	36.2%	59.6%	4.2%
35–49 years	734	35.2%	63.1%	1.8%
50–64 years	550	32.2%	66.9%	0.9%
65–79 years	321	31.2%	68.0%	0.8%
By BMI category				
Underweight	111	18.3%	61.2%	20.5%
Normal weight	1,032	31.0%	67.4%	1.6%
Overweight	643	33.9%	64.7%	1.4%
Obesity	408	43.2%	56.5%	0.3%
Children and adolescents (3–17 years)				
By sex				
Female	504	15.6%	81.8%	2.6%
Male	504	27.8%	70.5%	1.6%
By age group				
3–5 years	229	17.6%	77.0%	5.4%
6–8 years	214	17.9%	81.7%	0.4%
9–14 years	386	18.2%	80.1%	1.7%
15–17 years	179	36.5%	61.7%	1.8%
By BMI category				
Underweight	162	12.5%	86.5%	1.0%
Normal weight	637	20.3%	77.7%	2.0%
Overweight	146	29.5%	65.9%	4.6%
Obesity	63	42.4%	57.6%	0.0%

Percentages are weighted and sum to 100% across the three reporter categories within each stratum; *n* values are unweighted. BMI categories: WHO clinical cut-offs for adults; WHO/IOTF age- and sex-specific percentiles for children and adolescents. All associations with reporter status were significant (*χ*^2^, *p* < 0.001), except sex in adults (*p* = 0.049).

### 4.4 Strengths and limitations

A major strength of Nutrimetry lies in its continuous design and scalable online data-collection platform, which reduces operational costs and facilitates rapid implementation. The use of recipe decomposition to estimate raw-ingredient consumption represents an additional methodological innovation, enabling detailed assessment of adherence to national dietary recommendations such as the French Programme National Nutrition Santé (PNNS) (35, 36).

Several limitations should be acknowledged. First, the non-probability quota sampling design may limit generalisability despite post-stratification weighting. Second, dietary intake was self-reported and therefore subject to measurement error and under-reporting. Third, height and weight were also self-reported; because individuals with higher body weight tend to under-report weight and over-report height, and because basal metabolic rate is computed from these same self-reported anthropometrics, the observed association between under-reporting and higher BMI may partly reflect correlated measurement error rather than a fully independent relationship. Absolute intakes and the BMI–under-reporting association should therefore be interpreted with caution. Fourth, no biomarker validation (e.g., doubly labelled water or urinary markers) was available, and no relative-validity sub-study using an instrument with uncorrelated errors, such as a 24-h dietary recall, was conducted in the present wave or is currently planned; in principle, incorporating such a component in a subsample would be a valuable methodological addition, but it lies outside the scope of the present survey. Finally, the use of dietary supplements (vitamins or minerals) was not recorded, and clinical information—such as chronic conditions (e.g., diabetes, coeliac disease) or appetite- or metabolism-altering medication—was not collected; unrecorded supplement use may underestimate micronutrient intakes, and therapeutic diets can distort plausibility metrics. In addition, dietary records for children and adolescents were completed by a parent or legal guardian; while this ensured consistent administration and verification of consent across the 3–17-year sample, proxy reporting may be less accurate for adolescents aged 15–17 years, who usually self-report in comparable surveys, and could contribute to the higher under-reporting observed in that group. Adolescent self-completion is a possible refinement for future waves. Despite these limitations, Nutrimetry provides timely, nationally calibrated dietary data aligned with international standards and offers a sustainable framework for long-term nutrition surveillance in France.

## 5 Conclusion

Recent nationally representative data on dietary habits collected using rigorous and reproducible self-administered methods remain limited in France. The Nutrimetry survey addresses this gap by providing a scalable and continuous framework for dietary and nutritional surveillance. By generating updated data on food consumption patterns, nutrient intakes, and related lifestyle factors, Nutrimetry contributes valuable evidence to inform public health policies, nutrition education strategies, and food system initiatives. Continued implementation and methodological refinement will further strengthen its role in national nutrition monitoring.

## Data Availability

The datasets generated and analysed during the current study are not publicly available, but are available from the corresponding author on reasonable request. Requests to access the datasets should be directed to ES, elena.sauvage@c-ways.com.
