# Supplementary material for: Design and methodology of a continuous national dietary and nutrition survey in France: the Nutrimetry study
Source: Front Nutr. 2026 Aug 24;13:1909933. doi: 10.3389/fnut.2026.1909933 (PMC13547034; doi:10.3389/fnut.2026.1909933)
Supplement: Supplementary file 1 [file Data_Sheet_1.zip › Questionnaire Nutrimetry - English.PDF]

Supplementary Material

Nutrimetry study questionnaires (dietary record) — 2024

This document presents, as supplementary material, the two questionnaires administered electronically in Wave 1 (Q1 2024) of the Nutrimetry study: the recruitment/screening questionnaire and the post-record questionnaire, completed immediately after the three-day dietary record. The "Children" and "Adults" samples were surveyed through a single pathway, with questions specific to each subgroup.

The questionnaires were administered in French; the questions and response options reproduced below are an English translation provided for readability, the French original remaining the authoritative version. Hidden variables, post-hoc recoding, and the branching/conditional-display logic (used for questionnaire programming) have been removed for readability; only the questions actually asked are reported below.

Source: C-Ways / Potloc, Nutrimetry questionnaire, 2024.

Recruitment questionnaire

On-screen message: Welcome to this recruitment questionnaire for the Nutrimetry study!
To find out whether you can take part in this paid study ([reward amount]) on the eating habits of people in France, please answer the following questions (less than 3 minutes).

Sociodemographic data and screening questions

Q1. First, let's get to know each other.
What is your first name?

(Open answer)

On-screen message: Hello [first name]!
Nutrimetry is a large national survey conducted among people living in France. The aim of this survey is to understand your consumption habits and, who knows, to help improve the food of tomorrow!
The study will therefore take place over several days, and we are counting on your commitment to see it through to the end so that you can receive your reward of [reward amount] before 31 January 2025!
Here is the essential information about the Nutrimetry study and how it works:
It starts right now with 10 questions to find out who you are and whether you are eligible for the study.
You will then be invited to register on the "Nutrimetry" platform.
Next, on this platform, you will record your food consumption (all foods and beverages) over 3 days, which must meet the following rules:

- non-consecutive days,
- for adults over 18, a minimum of 750 kilocalories reported per day on at least 2 days is required (failure to meet this condition disqualifies you from the reward).
- if you are employed, at least one weekend day must be completed.
- if you work in a hybrid remote/on-site arrangement, at least one remote-working day and one on-site day must be completed.
- as representative as possible of your "normal" diet, on days when you were not following a diet or any other change to your usual way of eating.
- you have 2 weeks to record your consumption on the platform.

4️⃣Finally, to complete your participation, you will answer a 10-minute questionnaire.
All information provided in this study will be processed anonymously and for purely statistical purposes.

Q2. Interested in the study?

Yes, I am interested
No, let's stop here!

Q3. You are...?

A man
A woman

Q4. What is your age?

(Open answer: number)

Q5. What is the postcode of your place of residence?

Instruction: 5 digits maximum

(Open answer: number)

Q6. In which municipality do you live?

Instruction: This information will only be used to determine the type of area you live in (rural municipality; small, medium, or large urban area)

(Open answer)

Q7. How many people currently make up your household (including yourself)?

(Open answer: number)

Household composition and presence of children

Q8. Which family situation best describes the household you live in?

Couple with one or more children or parents
Couple without children

Single with one or more children or parents
Single without children
Other situations

Q9. Is an elderly person currently living in your household?

Yes
No

Q10. How many children are in your household?

No children
1
2
3
4
5
6
7
8

Q11. Please indicate their age and sex:
For each of them, please indicate their age and sex:

Instruction: For each child in the household concerned (from oldest to youngest):

First name
Age (0 to 99 years)
Sex (Male / Female)

Q12. Number of children by age and sex

Number of BOYS — aged 0 to 2: ___
Number of GIRLS — aged 0 to 2: ___
Number of BOYS — aged 3 to 5: ___
Number of GIRLS — aged 3 to 5: ___
Number of BOYS — aged 6 to 8: ___
Number of GIRLS — aged 6 to 8: ___
Number of BOYS — aged 9 to 14: ___
Number of GIRLS — aged 9 to 14: ___
Number of BOYS — aged 15 to 17: ___
Number of GIRLS — aged 15 to 17: ___
Number of BOYS — over 18: ___
Number of GIRLS — over 18: ___

Q13. Your child is eligible for the Nutrimetry study.
Would you agree that the foods and beverages consumed by your child be used for this study?

Yes
No

On-screen message: Congratulations! Your child [first name] is eligible for the Nutrimetry study.

Information on adults / parents

Q14. Back to you!
Are you currently employed?

Yes
No

Q15. Are you?

High-school student
University student
Retired
Looking for a first job
Previously employed and now looking for a job
Homemaker (Au foyer)
Other economically inactive: disabled, survivor's pension, etc.

Q16. What is your occupation?
What was your occupation?

Farmers (Agriculteurs)
Craftspeople, shopkeepers, business owners (Artisans, commerçants, chefs d'entreprise)
Senior managers and higher intellectual professions (Cadres supérieurs, professions intellectuelles supérieures)
Intermediate professions (Professions intermédiaires)
Clerical and service employees (Employés)
Manual workers (Ouvriers)

Q17. What is your employment status?

Private-sector employee on a permanent contract (CDI)
Private-sector employee on a fixed-term or other temporary contract (CDD)
Civil servant or public-sector employee on a permanent contract (fonctionnaire / CDI)
Public-sector employee on a fixed-term or other temporary contract (CDD)
Self-employed (including family workers) (Indépendant)
Other situations

Q18. What is the highest general or technical qualification you have obtained?

No qualification (Sans diplôme)
Primary-school certificate (Certificat d'études)
Lower-secondary certificate (Brevet / BEPC)

Vocational certificate (CAP / BEP)
High-school diploma (Bac)
Vocational high-school diploma (Bac professionnel)
2 years of higher education (Bac+2)
3 years of higher education / bachelor's (Bac+3)
4 years of higher education (Bac+4)
5 or more years of higher education / master's and above (Bac+5 et plus)

On-screen message: You have just told us about your personal situation.
We will now ask about the reference person of your household.

Q19. Do you think you are the reference person of your household?
The reference person is defined in the following order of priority:
- the oldest employed person with a partner;
- if no employed person has a partner, the oldest person with a partner;
- if no person has a partner, the oldest employed person;
- if no one is employed, the oldest person.

Yes
No, it is my (male) partner
No, it is my (female) partner
No, it is my father
No, it is my mother
No, it is another person (please specify:)

Q20. What is the sex of the reference person?

A man
A woman

Q21. What is the age of your [reference person]?

(Open answer: number)

Q22. Is your [reference person] currently employed?

Yes
No

Q23. Is your [reference person]...?

High-school student
University student
Retired
Looking for a first job
Previously employed and now looking for a job
Homemaker (Au foyer)
Other economically inactive: disabled, survivor's pension, etc.

Q24. What is their occupation?

Farmers (Agriculteurs)
Craftspeople, shopkeepers, business owners (Artisans, commerçants, chefs d'entreprise)
Senior managers and higher intellectual professions (Cadres supérieurs, professions intellectuelles supérieures)
Intermediate professions (Professions intermédiaires)
Clerical and service employees (Employés)
Manual workers (Ouvriers)

Q25. What is the highest general or technical qualification they have obtained?

No qualification (Sans diplôme)
Primary-school certificate (Certificat d'études)
Lower-secondary certificate (Brevet / BEPC)
Vocational certificate (CAP / BEP)
High-school diploma (Bac)
Vocational high-school diploma (Bac professionnel)
2 years of higher education (Bac+2)
3 years of higher education / bachelor's (Bac+3)
4 years of higher education (Bac+4)
5 or more years of higher education / master's and above (Bac+5 et plus)

Days off / working days (remote or on-site)

On-screen message: Let's continue with your own participation

Q26. Here is an attention-check question.
Which of these items is not a food product?

Pizza
Carrot
Eggs
Broccoli
Pen

Q27. In a typical working week, which are your days off?

Monday
Tuesday
Wednesday
Thursday
Friday
Saturday
Sunday

Q28. In a typical working week, how is your working time split between home and outside the home?

Items presented:

Monday
Tuesday
Wednesday
Thursday
Friday
Saturday
Sunday

Response options (for each item):

It is usually a day when I work remotely / from home
It is usually a day when I work away from home (e.g., at the company site, travelling, etc.)

Selection of the 3 dietary-record days

Q29. Assigned record day No. 1

(Open answer)

Q30. Assigned record day No. 2

(Open answer)

Q31. Assigned record day No. 3

(Open answer)

On-screen message: You will receive an email from us containing a link to a secure website where you will be able to:
1) Validate your participation agreement;
2) Register on the NUTRIMETRY website;
3) Access the diary in which to record your daily consumption.
You will also find information about this observatory and how to contact us.

On-screen message: Well done! You have been selected to take part in this study. Please follow this link to discover it.
Text: "To describe food consumption in all its complexity, we will ask you to complete a diary in which you record everything you consumed over three full days. After completing your diary on the dedicated website, you will be invited to return to our site to finalise the study. All information provided in this context will be processed anonymously and for purely statistical purposes."

Q32. Could you give us the email address you use most often? This is the address to which we will send the various pieces of information enabling you to take part in this study.

(Open answer)

Q33. Well done! You have been selected to take part in this study and are eligible to earn [reward amount] (electronic gift card) in exchange for your time!
Well done! Your child [child's first name] has been selected to take part in this study and is eligible to earn [reward amount] (electronic gift card) in exchange for your time!
Here are the next steps:
Provide your surname, first name, and email address (we will need them to send you the reward once the study is complete!) — Providing this information is entirely voluntary and you may withdraw your consent at any time.
Register on the Nutrimetry platform. When you register your child on this platform, please enter the child's own details (first name, age, sex) so that we know it is their consumption (and not yours)!
On this platform, you will record your child's food consumption over 3 days (15 to 20 minutes to complete per consumption day).
You have 2 weeks to complete the food consumption records on the platform.
At the end of the 3rd day, you will answer a 10-minute questionnaire.
Do you consent to provide this personal information and commit to completing the entire Nutrimetry study, and thereby earn an electronic gift card worth [reward amount] by 31 January 2025?
You have until 31 December 2024 to respond.

Yes
No

On-screen message: Well done! You have been selected to take part in this study!
Well done! Your child [child's first name] has been selected to take part in this study!
You have until 31 December 2024 to respond.

Q34. Please enter your first name, surname, and email address in the field(s) below. This information will allow us to contact you again to finalise your participation and to send you your reward at the end of the quarter.
To receive your reward of [reward amount] (electronic gift card), you will need to:
- correctly complete 3 days of food consumption on the Nutrimetry website;
- answer the final questionnaire.
The quality of your responses will be reviewed in the days following the end of your participation. If it meets our quality standards, you will receive your reward via an email from nutrimetrie@potloc.com. Don't forget to check your spam folder.
If you have any questions, please email us at nutrimetrie@potloc.com.
For more information and to exercise your rights, such as withdrawing your consent, please consult Potloc's Privacy Policy and Terms of Use.

(Open answer) Field(s) presented:

first name:
surname:
email address:

On-screen message: Click here to start registration on the Nutrimetry platform!
In a few seconds you will be automatically redirected to the Nutrimetry platform.
For information, you will need to record your child's diet on the following days:
- day 1: [day No. 1]
- day 2: [day No. 2]
- day 3: [day No. 3]

For information, you will choose yourself the 3 consumption days for your child to complete directly on the Nutrimetry platform.
For information, you will need to record your diet on the following days:
- day 1: [day No. 1]
- day 2: [day No. 2]
- day 3: [day No. 3]

For information, you will choose yourself the 3 consumption days to complete directly on the Nutrimetry platform, respecting the following rules:
- 3 non-consecutive days,
- for adults over 18, a minimum of 750 kilocalories reported per day on at least 2 days is required (failure to meet this condition disqualifies you from the reward).
- if you are employed, at least one weekend day must be completed.
- if you work in a hybrid remote/on-site arrangement, at least one remote-working day and one on-site day must be completed.
- as representative as possible of your "normal" diet, on days when you were not following a diet or any other change to your usual way of eating.
- you have 2 weeks to complete your food consumption on the platform.

Post-record questionnaire

On-screen message: Hello,
Thank you for the time you spent recording your diet over 3 days!
You have reached the final step of the Nutrimetry study, which will complete your participation: answering a 5- to 10-minute questionnaire.
Once this is done, we will contact you in the coming weeks by email to send you your reward*.
*To receive your reward of 15 euros before 31 January 2025, you must complete this final questionnaire.
The quality of your responses will be reviewed in the days following the end of your participation.
If it meets our quality standards, you will receive your reward. Don't forget to check your spam folder.

Section 1 — "Children" questionnaire

On-screen message: For all the following questions, parent and child, please both be present!

A — Record supplement

Q1. Were the three days your child has just recorded a weekday / weekend day / holiday, etc.?

Items presented:

The first recorded day
The second recorded day
The third recorded day

Response options (for each item):

A weekday, outside holidays
A weekend day or a public holiday
A holiday day

B — Physical activity, sleep, diet

Q2. Does your child take part in the school's physical-education classes?

Yes
No

Q3. In the past 7 days, on how many days did your child engage in vigorous physical activity (such as mountain biking or playing football)?

They had no vigorous physical activity this week
1 day
2 days
3 days
4 days
5 days
6 days
7 days

Q4. Does your child have difficulty falling asleep?

Yes
No
Don't know

Q5. After a typical night (outside holidays and weekends), how do they most often feel on waking?

Energetic
Rested
A little tired
Very tired
Don't know

Q6. Does your child eat at the school canteen at lunchtime?

Yes

No

Q7. How does your child rate the quality of the food served at the canteen?

Excellent
Good
Acceptable
Poor
Inadequate

C — Anthropometric data

Q8. What is your child's height?

Instruction: For example, 1 m 20; please enter 1.20

___ m

Q9. What is their weight?

Instruction: for example, 35 kg

___ kg

Q10. Is your child on a diet?
Do they follow a special diet?

Instruction: Multiple answers allowed

Yes, for medical reasons
Yes, they are vegetarian (i.e., they avoid meat and fish but consume eggs, cheese, or milk)
Yes, they are vegan (i.e., they avoid meat and fish, as well as all dairy products and eggs, and eat only grains, vegetables, and fruit)
Yes, they follow a lactose-free diet
Yes, they follow a gluten-free diet
Yes, they follow a diet because of allergies
Yes, to gain weight
Yes, to lose weight
Yes, to stay fit
Yes, for another reason (please specify)
No, they do not follow any particular diet

D — Child's nationality

On-screen message: A few more questions about your child.

Q11. The child selected to take part in the study is?

Of French origin

French by acquisition — acquired French nationality during their life
Of foreign origin
Prefer not to answer

Q12. What is their nationality of origin?
What is their nationality?

Algerian
Native American
Angolan
Belgian
Brazilian
British
Cameroonian
Canadian
Colombian
Congolese
Dominican
Egyptian
Spanish
Finnish
French
Gabonese
Haitian
Indian
Irish
Italian
Ivorian
Malagasy
Moroccan
Mauritian
Mauritanian
Polish
Portuguese
Romanian
Senegalese
Sri Lankan
Swiss
Togolese
Tunisian
Turkish
Yugoslav
Other (please specify)

Section 2 — "Adults" questionnaire

A — Record supplement

Q13. Were the three days you have just recorded a weekday / weekend day / holiday, etc.?

Items presented:

The first recorded day
The second recorded day
The third recorded day

Response options (for each item):

A weekday, outside holidays
A weekend day or a public holiday
A holiday day

Q14. Were the three days you have just recorded a day off, a remote-working day, or an on-site working day (at the company site, travelling, etc.)?

Items presented:

The first recorded day
The second recorded day
The third recorded day

Response options (for each item):

A day off
A day when I work remotely / from home
A day when I work away from home (e.g., at the company site, travelling, etc.)

Q15. Among the following products, please select the statement that best reflects your consumption during the survey.

Items presented:

Alcoholic beverages or wine
Processed/cured meats

Response options (for each item):

I consumed some during the days recorded in the diaries
I did not consume any during the recorded days, and I almost never consume them
I did not consume any during the recorded days, but I consume them occasionally
I did not consume any during the recorded days, but I consume them regularly

B — Physical activity, sleep, diet

On-screen message: We will now look at the different types of physical activity you do in your daily life.
Answer each of these questions even if you do not consider yourself to be an active person.
The questions concern the physical activity you do at work, in your home or garden, for getting around, and during your free time.

Q16. Think about all the vigorous activities you did in the past 7 days.
Definition: Vigorous physical activities are those that require a large physical effort and make you breathe much harder than normal. Consider only activities you did for at least 10 minutes at a time.
In the past 7 days, were there any days on which you did vigorous physical activities (fast running, walking faster than 6.5 km/h, climbing stairs briskly, cycling faster than 20 km/h, intense strength training, fast swimming, competitive sports and games such as football, volleyball, rugby, hockey, basketball, etc.)?

Yes (please specify how many days:)
No

Q17. In total, how much time did you spend on vigorous activities over the past 7 days?

Instruction: Specify the number of hours and minutes over the whole period (e.g., 12 h 30 over the past 7 days)

Number of hours: ___
Number of minutes: ___

Q18. Think about all the moderate activities you did in the past 7 days.
Definition: Moderate physical activities are those that require a moderate physical effort and make you breathe somewhat harder than normal. Consider only activities you did for at least 10 minutes at a time.
In the past 7 days, were there any days on which you did moderate physical activities (brisk walking between 5 and 6.5 km/h, dancing, cycling at 15 km/h, recreational swimming, gardening, housework and domestic chores, moderate strength training, etc.)?

Yes (please specify how many days:)
No

Q19. In total, how much time did you spend on moderate activities over the past 7 days?

Instruction: Specify the number of hours and minutes over the whole period (e.g., 10 h 30 over the past 7 days)

Number of hours: ___
Number of minutes: ___

Q20. Think about the time you spent walking in the past 7 days. This includes walking at work and at home, commuting, walking to go shopping, and any other walking you may have done in

your free time for relaxation, sport, or leisure.
In the past 7 days, were there any days on which you walked for at least 10 minutes at a time?

Yes (please specify how many days:)
No

Q21. In total, how much time did you spend walking (for at least 10 minutes at a time) over the past 7 days?

Instruction: Specify the number of hours and minutes over the whole period (e.g., 5 h 30 over the past 7 days)

Number of hours: ___
Number of minutes: ___

Q22. In the past 7 days, how much time did you spend sitting on an average weekday (excluding weekends)?

Instruction: Specify the number of hours and minutes on a typical day

Number of hours per day: ___
Number of minutes per day: ___

Q23. On average, how many hours do you sleep per day?

Instruction: "Per day" means "from midnight to midnight". Specify the number of hours and minutes

Number of hours per night: ___
Number of minutes per night: ___

Q24. After a typical night (outside holidays and weekends), how do you most often feel on waking?

Energetic
Rested
A little tired
Very tired

Q25. Do you smoke (tobacco)?

No
Yes, occasionally
Yes, regularly
I am in the process of quitting

Q26. Are you currently pregnant?

Yes

No
Not applicable

On-screen message: Let's now look at your food purchasing and consumption habits.

Q27. In your household, who most often does the food shopping?

Myself
My partner
My father
My mother
Another person

Q28. In your household, who most often does the cooking?

Myself
My partner
My father
My mother
Another person

Q29. Here are several questions about the use of fats in your diet.
Which fats do you use most often for the different purposes listed?

Items presented:

For cooking meat in a pan or in the oven
For cooking eggs as an omelette or fried
When serving meat
For cooking fish in a pan or in the oven
When serving fish
For cooking vegetables in a pan
For cooking rice or pasta
When serving vegetables
When serving starchy foods (rice, pasta, etc.)
For cooking chips/fries
For dressing raw vegetables

Response options (for each item):

I do not use any fat for this purpose / I do not eat this dish
01. unsalted, full-fat butter
02. salted, full-fat butter
03. reduced-fat butter
04. regular margarine, 70% or 80% fat
05. sunflower margarine, 70% or 80% fat
06. regular margarine, 60% fat

07. olive-oil margarine, 60% fat
08. sunflower margarine, 60% fat
09. "Primevère"-type margarine, 60% fat
10. reduced-fat margarine, 40% fat
11. reduced-fat semi-salted margarine, 40% fat
12. "Pro-activ" margarine (Fruit d'Or)
13. solid vegetable cooking fat (Végétaline)
14. full-fat crème fraîche
15. reduced-fat crème fraîche
16. peanut oil
17. blended oil (e.g., Isio 4)
18. sunflower oil
19. olive oil
20. rapeseed (canola) oil
21. corn oil
22. other oil
23. beef fat, lard, or other animal fat
24. mayonnaise
25. vinaigrette
26. reduced-fat vinaigrette
27. other, please specify

Q30. Do you sometimes have people over for lunch or dinner at your home?

Yes, regularly
Yes, occasionally
No

Q31. In the past 12 months, has your household ever reduced the size of meals or skipped meals because there was not enough money for food?

Yes
No

Q32. How often did this happen?

Almost every month
Some months but not every month
Only once or twice during the year

Q33. In the past 12 months, did you personally ever eat less than you felt you should because there was not enough money to buy food?

Yes
No

Q34. In the past 12 months, were you personally ever hungry but did not eat because you could not afford enough food?

Yes
No

Q35. Please tell us whether the following situations happened to you in the past 12 months.

Items presented:

The food we bought did not last, and there was not enough money to buy more.
It is difficult (financially) to eat balanced meals.

Response options (for each item):

It happened often
It happened sometimes
It never happened

Q36. At your workplace, do you have access to...?

Instruction: Multiple answers allowed

A staff canteen (restaurant d'entreprise)
A dedicated area at your workplace (with a table, a fridge, and/or an oven available)
Meal vouchers (titres-restaurant: Ticket Restaurant, Chèques Déjeuner, Chèques Restaurant, Chèques de Table, Carte Chèque Déjeuner)
None of these

Q37. What is the value of your meal vouchers (titres-restaurant) for one working day?

(Open answer) Field(s) presented:

__ euros __ cents

Q38. Do you use your vouchers (titres-restaurant) mainly for lunch on working days?

Yes
No

Q39. In which types of outlet do you use your meal vouchers (titres-restaurant)?

Instruction: Multiple answers allowed

In restaurants
In a staff canteen (restaurant d'entreprise)
In a bakery / takeaway
In a supermarket, grocery, or fruit-and-vegetable shop

Q40. During the 3 survey days, did you use a meal voucher (ticket-restaurant)?

Instruction: Multiple answers allowed

Yes, on the first recorded day
Yes, on the second recorded day
Yes, on the third recorded day
No, I did not use any during the 3 survey days

Q41. What is your household's weekly food budget for meals eaten at home, including drinks (excluding meals eaten out)?

(Open answer) Field(s) presented:

__ euros

Q42. Are you concerned about the following issues relating to your diet?

Items presented:

The quality of food products
Animal welfare
Environmental impact
Fair remuneration of producers
Transparency about the origin of products

Response options (for each item):

Very concerned
Somewhat concerned
Not really concerned
Not at all concerned

C — Anthropometric data and lifestyle

Q43. What is your height?

Instruction: For example, 1 m 70; please enter 1.70

___ m

Q44. What is your weight?

Instruction: for example, 70 kg

___ kg

Q45. Are you on a diet?
Do you follow a special diet?

Instruction: Multiple answers allowed

Yes, for medical reasons

Yes, I am vegetarian (i.e., I avoid meat and fish but consume eggs, cheese, or milk)
Yes, I am vegan (i.e., I avoid meat and fish, as well as all dairy products and eggs, and eat only grains, vegetables, and fruit)
Yes, I follow a lactose-free diet
Yes, I follow a gluten-free diet
Yes, I follow a diet because of allergies
Yes, to gain weight
Yes, to lose weight
Yes, to stay fit
Yes, for another reason (please specify)
No, I do not follow any particular diet

D — Adult's nationality / cultural influences

On-screen message: A few more questions about you.

Q46. Are you?

Of French origin
French by acquisition — acquired French nationality during their life
Of foreign origin
Prefer not to answer

Q47. What is your nationality of origin?
What is your nationality?

Algerian
Native American
Angolan
Belgian
Brazilian
British
Cameroonian
Canadian
Colombian
Congolese
Dominican
Egyptian
Spanish
Finnish
French
Gabonese
Haitian
Indian
Irish
Italian
Ivorian

Malagasy
Moroccan
Mauritian
Mauritanian
Polish
Portuguese
Romanian
Senegalese
Sri Lankan
Swiss
Togolese
Tunisian
Turkish
Yugoslav
Other (please specify)
Prefer not to answer

Q48. During 2023, did you ever eat...?

Instruction: Multiple answers allowed

Halal products
Kosher products
Neither
Prefer not to answer

Q49. During 2023, how often did you eat halal products?

Instruction: Multiple answers allowed

More than once a week
Once a week
Two to three times a month
Once a month
Less often
Don't know

Q50. During 2023, how often did you eat kosher products?

Instruction: Multiple answers allowed

More than once a week
Once a week
Two to three times a month
Once a month
Less often
Don't know

Q51. Regarding religion and spirituality, would you say you are?

Instruction: Multiple answers allowed

A believer and practising
A believer but not practising
Not interested in these matters

E — Household information (ALL RESPONDENTS)

On-screen message: Almost done! A few more details about your household.

Q52. Do you live in...?

A detached/individual house
A flat in a building or multi-unit dwelling

Q53. Is your household...?

Renting
Owner or buying (with a mortgage)
Housed free of charge

Q54. Do you have a garden where you grow fruit or vegetables?

Yes
No

Q55. Among the following situations, which have already happened to you?

Instruction: Multiple answers allowed

Not having a personal car for financial reasons
Not being able to keep your home at a comfortable temperature for financial reasons
Not being able to replace worn-out furniture for financial reasons
Having unpaid loan instalments, rent, or electricity, water, or gas bills
Not being able to spend a small amount of money on yourself without having to consult anyone
Not being able to cope with unexpected expenses
Not having internet access for financial reasons
Not being able to meet friends or family at least once a month for a drink or a meal for financial reasons
Not having a regular leisure activity for financial reasons
Not being able to afford a week's holiday away from home for financial reasons
Not having two pairs of good shoes for financial reasons
Not being able to have a meal containing protein at least every other day for financial reasons
Not being able to buy new clothes
None of these situations has happened to me

Q56. Which of the following statements best describes your household's current financial situation?

- You have taken out one (or more) loans to make ends meet
- You are drawing somewhat on your savings
- You just balance your budget with your income
- You manage to set aside a little money
- You manage to set aside a fair amount of money

Q57. Please indicate the bracket that broadly corresponds to your household income.

Instruction: That is, your personal income and, where applicable, that of your partner (before tax and after social contributions, all income combined: wages, bonuses, benefits, pensions, financial income, etc.)

- Less than €1,000 / MONTH (or less than €12,000 / YEAR)
- €1,000–1,199 / MONTH (or €12,000–14,399 / YEAR)
- €1,200–1,399 / MONTH (or €14,400–16,799 / YEAR)
- €1,400–1,699 / MONTH (or €16,800–20,399 / YEAR)
- €1,700–1,999 / MONTH (or €20,400–23,999 / YEAR)
- €2,000–2,299 / MONTH (or €24,000–27,599 / YEAR)
- €2,300–2,999 / MONTH (or €27,600–35,999 / YEAR)
- €3,000–4,499 / MONTH (or €36,000–53,999 / YEAR)
- €4,500–5,999 / MONTH (or €54,000–71,999 / YEAR)
- €6,000 or more / MONTH (or €72,000 or more / YEAR)
- Don't know
- Prefer not to answer

On-screen message: Thank you for taking part in the Nutrimetry survey. You have reached the end.
The quality of your responses will be reviewed in the coming days/weeks. If it meets our quality standards, you will receive your reward via an email from nutrimetrie@potloc.com, no later than 31 January 2025. Don't forget to check your spam folder.
If you have any questions, please email us at nutrimetrie@potloc.com.
