# Supplementary material for: Design and methodology of a continuous national dietary and nutrition survey in France: the Nutrimetry study
Source: Front Nutr. 2026 Aug 24;13:1909933. doi: 10.3389/fnut.2026.1909933 (PMC13547034; doi:10.3389/fnut.2026.1909933)
Supplement: Supplementary file 1 [file Data_Sheet_1.zip › Questionnaire Nutrimetry - French.PDF]

Matériel supplémentaire

Questionnaires de l'étude Nutrimétrie (carnet alimentaire) — Vague 2024

Ce document présente, à titre de matériel supplémentaire, les deux questionnaires administrés par voie électronique dans le cadre de la vague 4 (T4 2024) de l'étude Nutrimétrie : le questionnaire de recrutement / screening et le questionnaire post-carnet, complété immédiatement après les trois jours de relevé alimentaire. Les échantillons "Enfants" et "Adultes" ont été interrogés au moyen d'un même parcours, avec des questions spécifiques à chaque sous-groupe.

Les questions et modalités de réponse reproduisent le libellé soumis aux répondants. Les variables cachées, les recodages a posteriori et la logique de branchement / d'affichage conditionnel (utilisés pour la programmation du questionnaire) ont été retirés par souci de lisibilité ; seules les questions effectivement posées sont reportées ci-dessous.

Source : C-Ways / Potloc, questionnaire Nutrimétrie, 2024.

Questionnaire de recrutement

Message affiché à l'écran : Bienvenue sur ce questionnaire de recrutement à l'étude Nutrimétrie ! Pour savoir si vous pouvez participer à cette étude rémunérée ([montant de la récompense]) sur les habitudes alimentaires des Français(es), merci de répondre aux questions suivantes (moins de 3 minutes).

Données sociodémographiques et questions de sélection

Q1. Tout d'abord pour commencer, faisons connaissance.
Quel est votre prénom ?

(Réponse libre)

Message affiché à l'écran : Bonjour [prénom] !
Nutrimétrie est une grande enquête nationale réalisée auprès de personnes vivant en France.
L'objectif de cette enquête est de comprendre vos habitudes de consommation, et qui sait, améliorer l'alimentation de demain !
Cette étude va donc avoir lieu sur une durée de plusieurs jours, et nous comptons sur votre engagement pour aller jusqu'au bout afin de recevoir votre récompense de [montant de la récompense] avant le 31 janvier 2025 !
Voici les informations à savoir absolument sur l'étude Nutrimétrie et son déroulé :
Ça commence dès à présent par 10 questions pour savoir qui vous êtes et si vous êtes éligible à l'étude.
Puis, vous serez invité(e) à vous inscrire sur la plateforme "Nutrimétrie".
Ensuite, sur cette plateforme, vous remplirez vos consommations alimentaires (tous les aliments et boissons) durant 3 jours qui doivent respecter ces règles :

- non consécutifs,
- pour les adultes de plus de 18 ans un nombre minimum de 750 kilocalories déclarées par jour sur au moins 2 jours est exigé (le non respect de cette condition est éliminatoire à l'accès à la récompense).
- si vous exercez une activité professionnelle, au moins un jour de week-end doit être rempli.
- si vous exercez une activité professionnelle en mode hybride télétravail/présentiel, au moins un jour de télétravail et un jour de présentiel doivent être remplis.
- les plus représentatifs possible de votre alimentation "normale" et durant lesquels vous n'avez pas suivi de régime ou autre changement dans la façon habituelle de vous alimenter.
- vous disposez de 2 semaines pour remplir votre consommation sur la plateforme.

4 Enfin, pour clôturer votre participation, vous répondrez à un questionnaire de 10 minutes.
Toutes les informations communiquées dans le cadre de cette étude seront traitées de façon anonyme et à des fins purement statistiques.

Q2. Intéressé(e) par l'étude ?

Oui, je suis intéressé(e)
Non, arrêtons dès à présent !

Q3. Vous êtes...?

Un homme
Une femme

Q4. Quel est votre âge ?

(Réponse libre : nombre)

Q5. Quel est le code postal de votre lieu de résidence ?

Consigne : 5 chiffres maximum

(Réponse libre : nombre)

Q6. Dans quelle commune vivez-vous ?

Consigne : Cette information permettra uniquement de savoir dans quel type d'habitat vous vivez (commune rurale, petite, moyenne ou grande agglomération)

(Réponse libre)

Q7. Combien de personnes composent aujourd’hui votre foyer (vous y compris) ?

(Réponse libre : nombre)

Composition du foyer et présence d'enfants

Q8. Quelle situation familiale décrit le mieux le foyer dans lequel vous vivez ?

Couple avec un ou plusieurs enfants ou parents

Couple sans enfants
Seul(e) avec un ou plusieurs enfants ou parents
Seul(e) sans enfant
Autres situations

Q9. Une personne âgée est-elle actuellement présente au sein de votre foyer ?

Oui
Non

Q10. Combien d'enfants composent votre foyer ?

Aucun enfant
1
2
3
4
5
6
7
8

Q11. Pouvez-vous nous indiquer son âge et son sexe :
Pour chacun d'entre eux, pouvez-vous nous indiquer son âge et son sexe :

Consigne : Pour chaque enfant du foyer concerné (dans l'ordre du plus âgé au plus jeune) :

Prénom
Âge (0 à 99 ans)
Genre (Masculin / Féminin)

Q12. Nombre d'enfant(s) par âge et sexe

Nombre de GARÇONS - entre 0 et 2 ans : ___
Nombre de FILLES - entre 0 et 2 ans : ___
Nombre de GARÇONS - entre 3 et 5 ans : ___
Nombre de FILLES - entre 3 et 5 ans : ___
Nombre de GARÇONS - entre 6 et 8 ans : ___
Nombre de FILLES - entre 6 et 8 ans : ___
Nombre de GARÇONS - entre 9 et 14 ans : ___
Nombre de FILLES - entre 9 et 14 ans : ___
Nombre de GARÇONS - entre 15 et 17 ans : ___
Nombre de FILLES - entre 15 et 17 ans : ___
Nombre de GARÇONS - plus de 18 ans : ___
Nombre de FILLES - plus de 18 ans : ___

Q13. Votre enfant est éligible à l'étude Nutrimétrie.
Accepteriez-vous que ce soient les aliments et boissons consommés par votre enfant qui soient utilisés pour cette étude ?

Oui
Non

Message affiché à l'écran : Félicitations ! Votre enfant [prénom] est éligible à l'étude Nutrimétrie.

Informations sur les adultes / parents

Q14. Revenons à vous !
Actuellement, exercez-vous une activité professionnelle ?

Oui
Non

Q15. Êtes-vous ?

Lycéen(ne)
Étudiant(e)
Retraité(e)
À la recherche d'un premier emploi
Vous avez déjà travaillé et vous êtes à la recherche d'un emploi
Au foyer
Autre inactif : invalide, pension de réversion…

Q16. Quelle est votre profession ?
Quelle était votre profession ?

Agriculteurs
Artisans - Commerçants - Chefs d'entreprise
Cadres supérieurs - Professions intellectuelles supérieures
Professions intermédiaires
Employés
Ouvriers

Q17. Quel est votre statut professionnel ?

Salarié en CDI du secteur privé
Salarié en CDD ou autre contrat à durée déterminée du secteur privé
Fonctionnaire ou contractuel en CDI de la fonction publique
Contractuel en CDD ou autre contrat à durée déterminée de la fonction publique
Indépendante (y compris aide familial)
Autres situations

Q18. Quel est le diplôme d'enseignement général ou technique le plus élevé que vous ayez obtenu ?

Sans diplôme
Certificat d’études
Brevet / BEPC
CAP / BEP
Bac
Bac Professionnel
Bac+2
Bac+3
Bac+4
Bac+5 et plus

Message affiché à l'écran : Vous venez de nous indiquer votre situation personnelle. Nous allons nous intéresser à la personne de référence de votre foyer.

Q19. Pensez-vous être la personne de référence de votre foyer ?
La personne de référence est définie par ordre de priorité suivante :
- la personne active la plus âgée ayant un conjoint ;
- si pas de personne active ayant un conjoint, la personne la plus âgée ayant un conjoint ;
- si pas de personne ayant un conjoint, la personne active la plus âgée ;
- si pas de personne active, la personne la plus âgée.

Oui
Non, c'est mon conjoint
Non, c'est ma conjointe
Non, c'est mon père
Non, c'est ma mère
Non, c'est une autre personne (précisez :)

Q20. Quel est le sexe de la personne de référence ?

Un homme
Une femme

Q21. Quel est l'âge de votre [personne de référence] ?

(Réponse libre : nombre)

Q22. Actuellement, votre [personne de référence] exerce-t-il une activité professionnelle ?
Actuellement, votre [personne de référence] exerce-t-elle une activité professionnelle ?

Oui
Non

Q23. Votre [personne de référence] est-il...?
Votre [personne de référence] est-elle...?

Lycéen(ne)
Étudiant(e)

Retraité(e)
À la recherche d'un premier emploi
Vous avez déjà travaillé et vous êtes à la recherche d'un emploi
Au foyer
Autre inactif : invalide, pension de réversion…

Q24. Quelle est sa profession ?

Agriculteurs
Artisans - Commerçants - Chefs d'entreprise
Cadres supérieurs - Professions intellectuelles supérieures
Professions intermédiaires
Employés
Ouvriers

Q25. Quel est le diplôme d'enseignement général ou technique le plus élevé qu'il / elle a obtenu ?

Sans diplôme
Certificat d'études
Brevet / BEPC
CAP / BEP
Bac
Bac Professionnel
Bac+2
Bac+3
Bac+4
Bac+5 et plus

Jours de repos / jours travaillés (télétravail ou non)

Message affiché à l'écran : Poursuivons donc avec votre propre participation

Q26. Voici une question d'attention.
Lequel de ces produits n'est pas un produit alimentaire ?

Pizza
Carotte
Œufs
Brocolis
Stylo

Q27. Dans une semaine habituelle travaillée, quels sont vos jours de repos ?

Lundi
Mardi
Mercredi
Jeudi
Vendredi

Samedi
Dimanche

Q28. Dans une semaine habituelle travaillée, comment se répartit votre temps de travail à la maison versus en dehors de chez vous ?

Éléments proposés :

Lundi
Mardi
Mercredi
Jeudi
Vendredi
Samedi
Dimanche

Modalités de réponse (pour chaque élément) :

C'est habituellement un jour où je télé-travaille / ou je travaille chez moi
C'est habituellement un jour où je travaille en dehors de chez moi (par exemple : sur le site de l'entreprise, en déplacement...)

Sélection des 3 jours de relevé alimentaire

Q29. Jour de relevé imposé n°1

(Réponse libre)

Q30. Jour de relevé imposé n°2

(Réponse libre)

Q31. Jour de relevé imposé n°3

(Réponse libre)

Message affiché à l'écran : Vous allez recevoir un email de notre part contenant un lien pour vous rendre sur un site Internet sécurisé où vous pourrez :
1) Valider votre convention de participation;
2) Vous inscrire sur le site NUTRIMETRIE;
3) Accéder au carnet où noter vos consommations quotidiennes.
Vous y trouverez aussi des informations sur cet observatoire et les moyens de nous contacter.

Message affiché à l'écran : Bravo ! Vous êtes sélectionné pour participer à cette étude. Veuillez suivre ce lien pour la découvrir.
Proposition : « Pour décrire dans toute sa complexité la consommation alimentaire nous allons vous demander de remplir un carnet sur lequel vous indiquerez tout ce que vous avez consommé sur trois journées complètes. Après avoir rempli votre carnet sur le site Internet dédié, vous serez

invité à revenir sur notre site pour finaliser l'étude. Toutes les informations communiquées dans ce cadre seront traitées de façon anonyme et à des fins purement statistiques. »

Q32. Pouvez-vous me communiquer l'adresse email que vous utilisez le plus ? C'est à cette adresse email que nous allons vous faire parvenir les différents éléments d'information vous permettant de participer à cette étude.

(Réponse libre)

Q33. Bravo ! Vous êtes sélectionné(e) pour participer à cette étude et éligible pour gagner [montant de la récompense] (carte cadeau électronique) en échange de votre temps !
Bravo ! Votre enfant [prénom de l'enfant] est sélectionné pour participer à cette étude et éligible pour gagner [montant de la récompense] (carte cadeau électronique) en échange de votre temps !
Voici les prochaines étapes :
Indiquez vos nom, prénom et adresse électronique (nous en aurons besoin pour vous communiquer la récompense une fois l'étude terminée !) - La fourniture de ces informations est entièrement volontaire et vous pouvez révoquer votre consentement à tout moment.
Inscrivez-vous à la plateforme Nutrimétrie. Lorsque vous inscrirez votre enfant sur cette plateforme, merci d'indiquer ses coordonnées à lui (prénom, âge, sexe de votre enfant) pour que nous puissions savoir que c'est sa consommation (et non la vôtre) !
Sur cette plateforme, vous remplirez vos consommations alimentaires les consommations alimentaires de votre enfant durant 3 jours (15 à 20 min de remplissage par jour de consommation).
Vous disposez de 2 semaines pour compléter vos consommations alimentaires sur la plateforme.
À la clôture du 3ème jour, vous répondrez à un questionnaire de 10 minutes.
Consentez-vous à fournir ces informations personnelles et engagez-vous à compléter l'ensemble de l'étude Nutrimétrie et ainsi gagner une carte cadeau électronique d'un montant de [montant de la récompense] d'ici le 31 janvier 2025 ?
Vous avez jusqu'au 31 décembre 2024 pour répondre.

Oui
Non

Message affiché à l'écran : Bravo ! Vous êtes sélectionné(e) pour participer à cette étude !
Bravo ! Votre enfant [prénom de l'enfant] est sélectionné pour participer à cette étude !
Vous avez jusqu'au 31 décembre 2024 pour répondre.

Q34. Veuillez saisir vos prénom, nom et adresse électronique dans le(s) champ(s) ci-dessous. Ces informations nous permettront de vous recontacter afin de finaliser votre participation et de vous envoyer votre récompense à l'issue du trimestre
Pour recevoir votre récompense d'un montant de [montant de la récompense] (carte cadeau électronique), vous devrez :
- compléter correctement 3 jours de consommation alimentaire sur le site Nutrimétrie
- répondre au dernier questionnaire.
La qualité de vos réponses sera examinée dans les jours qui suivent la fin de votre participation.
Si elle répond à nos normes de qualité, vous recevrez votre récompense via un e-mail de

nutrimetrie@potloc.com. N'oubliez pas de consultez vos spams.
Si vous avez des questions, veuillez nous envoyer un email à nutrimetrie@potloc.com.
Pour de plus amples informations et pour exercer vos droits tels que la révocation de votre consentement, veuillez consulter la Politique de Confidentialité et les Conditions d'Utilisation de Potloc.

(Réponse libre) Champ(s) proposé(s) :

prénom :
nom :
adresse électronique :

Message affiché à l'écran : Cliquez ici pour démarrer l'inscription sur la plateforme Nutrimétrie !
Dans quelques secondes, vous allez être redirigé(e) automatiquement sur la plateforme Nutrimétrie.
Pour information, vous devrez renseigner l'alimentation de votre enfant les jours suivants :
- jour 1 : [jour n°1]
- jour 2 : [jour n°2]
- jour 3 : [jour n°3]
Pour information, vous choisirez vous-même les 3 journées de consommation de votre enfant à remplir directement sur la plateforme Nutrimétrie.
Pour information, vous devrez renseigner votre alimentation les jours suivants :
- jour 1 : [jour n°1]
- jour 2 : [jour n°2]
- jour 3 : [jour n°3]
Pour information, vous choisirez vous-même les 3 journées de consommation à remplir directement sur la plateforme Nutrimétrie, en respectant les consignes suivantes :
- 3 jours non consécutifs,
- pour les adultes de plus de 18 ans un nombre minimum de 750 kilocalories déclarées par jour sur au moins 2 jours est exigé (le non respect de cette condition est éliminatoire à l'accès à la récompense).
- si vous exercez une activité professionnelle, au moins un jour de week-end doit être rempli.
- si vous exercez une activité professionnelle en mode hybride télétravail/présentiel, au moins un jour de télétravail et un jour de présentiel doivent être remplis.
- les plus représentatifs possible de votre alimentation "normale" et durant lesquels vous n'avez pas suivi de régime ou autre changement dans la façon habituelle de vous alimenter.
- vous disposez de 2 semaines pour compléter votre consommation alimentaire sur la plateforme.

Questionnaire post-carnet

Message affiché à l'écran : Bonjour,
Nous vous remercions pour votre temps à renseigner votre alimentation durant 3 jours !
Vous arrivez à la dernière étape de l'étude Nutrimétrie qui clôturera votre participation : répondre

à un questionnaires de 5 à 10 minutes.
Une fois que ce sera fait, nous vous recontacterons dans les prochaines semaines via votre courriel pour vous envoyer votre récompense*.
*Pour recevoir votre récompense de 15 euros avant le 31 janvier 2025, vous devrez compléter ce dernier questionnaire.
La qualité de vos réponses sera examinée dans les jours qui suivent la fin de votre participation. Si elle répond à nos normes de qualité, vous recevrez votre récompense. N'oubliez pas de consultez vos spams.

Section 1 - Questionnaire "Enfants"

Message affiché à l'écran : Pour l'ensemble des prochaines questions, parent + enfant, merci d'être présents tous les 2 !

A- Complément au carnet

Q1. Les trois jours que votre enfant vient de remplir étaient-ils un jour de semaine / de week-end, de vacances...?

Éléments proposés :

Le premier jour de remplissage
Le deuxième jour de remplissage
Le troisième jour de remplissage

Modalités de réponse (pour chaque élément) :

Un jour de semaine, hors vacances
Un jour de week-end ou un jour férié
Un jour de vacances

B- Activité physiqué, Sommeil, Alimentation

Q2. Votre enfant participe-t-il aux cours de sport de l'école ?

Oui
Non

Q3. Au cours des 7 derniers jours, combien y a-t-il eu de jours au cours desquels votre enfant a fait des activités physiques intenses (comme faire du VTT ou jouer au football) ?

Il n'a pas eu d'activité physique intense cette semaine
1 jour
2 jours
3 jours
4 jours
5 jours
6 jours
7 jours

Q4. Votre enfant connaît-il des difficultés à s'endormir ?

Oui
Non
Je ne sais pas

Q5. Après une nuit habituelle (hors vacances et week-end), comment se sent-il le plus souvent au réveil ?

En forme
Reposé
Un peu fatigué
Très fatigué
Je ne sais pas

Q6. Votre enfant va-t-il à la cantine le midi ?

Oui
Non

Q7. Comment votre enfant juge-t-il la qualité de la nourriture proposée à la cantine ?

Excellente
Bonne
Correcte
Médiocre
Insuffisante

C- Données anthropométriques

Q8. Quelle taille fait votre enfant ?

Consigne : Par exemple : 1m20, merci de noter : 1,20

___ m

Q9. Quel est son poids ?

Consigne : par exemple 35 kg

___ kg

Q10. Votre enfant fait-il un régime ?
A-t-il une alimentation particulière ?

Consigne : Plusieurs réponses possibles

Oui, pour raisons médicales
Oui, il ou elle est végétarien(ne) (c'est-à-dire qu'il ou elle s'abstient de manger de la viande, du poisson, mais consomme des œufs, du fromage ou du lait)

Oui, il ou elle est végan ou végétalien(ne) (c'est-à-dire qu'il s'abstient de manger de la viande, du poisson, mais aussi tous les produits laitiers et les œufs, et ne mange que des céréales, des légumes et des fruits)
Oui, il ou elle a un régime sans lactose
Oui, il ou elle a un régime sans gluten
Oui, il ou elle a un régime en raison d'allergies
Oui, pour prendre du poids
Oui, pour maigrir
Oui, pour rester en forme
Oui, pour une autre raison (précisez laquelle)
Non, il ou elle ne suit aucun régime particulier

D- Nationalité de l'enfant

Message affiché à l'écran : Encore quelques questions concernant votre enfant.

Q11. L'enfant sélectionné pour participer à l'étude est-il ?

D'origine française
Français(e) d'acquisition – a acquis la nationalité française au cours de sa vie
D'origine étrangère
Je ne veux pas répondre

Q12. Quelle est sa nationalité d'origine ?
De quelle nationalité est-il ?

Algérienne
Amérindienne
Angolaise
Belge
Brésilienne
Britannique
Camerounaise
Canadienne
Colombienne
Congolaise
Dominicaine
Égyptienne
Espagnole
Finlandaise
Française
Gabonaise
Haitienne
Indienne
Irlandaise
Italienne
Ivoirienne

Malgache
Marocaine
Mauricienne
Mauritanienne
Polonaise
Portugaise
Roumaine
Sénégalaise
Sri-Lankaise
Suisse
Togolaise
Tunisienne
Turque
Yougoslave
Autre (précisez)

Section 2 - Questionnaire "Adultes"

A- Complément au carnet

Q13. Les trois jours que vous venez de remplir étaient-ils un jour de semaine / de week-end, de vacances...?

Éléments proposés :

Le premier jour de remplissage
Le deuxième jour de remplissage
Le troisième jour de remplissage

Modalités de réponse (pour chaque élément) :

Un jour de semaine, hors vacances
Un jour de week-end ou un jour férié
Un jour de vacances

Q14. Les trois jours que vous venez de remplir étaient-ils un jour de repos, de télétravail, de travail sur site (sur le site de l'entreprise, en déplacement...) ?

Éléments proposés :

Le premier jour de remplissage
Le deuxième jour de remplissage
Le troisième jour de remplissage

Modalités de réponse (pour chaque élément) :

Un jour de repos
C'est un jour où je télé-travaille / ou je travaille chez moi

C'est un jour où je travaille en dehors de chez moi (par exemple : sur le site de l'entreprise, en déplacement...)

Q15. Parmi les produits suivants, pouvez-vous sélectionner la phrase qui représente le mieux votre consommation pendant l'enquête ?

Éléments proposés :

Des boissons alcoolisées ou du vin
Des charcuteries

Modalités de réponse (pour chaque élément) :

J’en ai consommé pendant les jours décrits sur les carnets
Je n’en ai pas consommé pendant les jours décrits sur les carnets, et je ne n’en consomme jamais ou presque
Je n’en ai pas consommé pendant les jours décrits sur les carnets, mais j’en consomme de temps en temps
Je n’en ai pas consommé pendant les jours décrits sur les carnets, mais j’en consomme régulièrement

B- Activité physiqué, Sommeil, Alimentation

Message affiché à l'écran : Nous allons nous intéresser aux différents types d’activités physiques que vous faites dans votre vie quotidienne.
Répondez à chacune de ces questions même si vous ne vous considérez pas comme une personne active.
Les questions concernent les activités physiques que vous faites au travail, dans votre maison ou votre jardin, pour vos déplacements, et pendant votre temps libre.

Q16. Pensez à toutes les activités intenses que vous avez faites au cours des 7 derniers jours.
Définition : Les activités physiques intenses font référence aux activités qui vous demandent un effort physique important et vous font respirer beaucoup plus difficilement que normalement.
Pensez seulement aux activités que vous avez effectuées pendant au moins 10 minutes d’affilée.
Au cours des 7 derniers jours, y a-t-il eu des jours au cours desquels vous avez pratiqué des activités physiques intenses (course rapide, marche à plus de 6,5 km/h), monter des escaliers à vive allure, vélo à plus de 20 km/h, renforcement musculaire intense, natation à vive allure, sports et de jeux de compétition comme le football, volleyball, rugby, hockey, basket…) ?

Oui (précisez combien de jours :)
Non

Q17. Au total, combien de temps avez-vous passé à faire des activités intenses au cours des 7 derniers jours ?

Consigne : Précisez le nombre d'heures et minutes sur l'ensemble de la période (par exemple : 12 h 30 sur les 7 derniers jours)

Nombre d'heures : ___

Nombre de minutes : ___

Q18. Pensez à toutes les activités modérées que vous avez faites au cours des 7 derniers jours.
Définition : Les activités physiques modérées font référence aux activités qui vous demandent un effort physique modéré et vous font respirer un peu plus difficilement que normalement. Pensez seulement aux activités que vous avez effectuées pendant au moins 10 minutes d'affilée.
Au cours des 7 derniers jours, y a-t-il eu des jours au cours desquels vous avez pratiqué des activités physiques modérées (marche d'un pas vif entre 5 et 6,5 km/h, danse, vélo à 15 km/h, nage de loisir, jardinage, travaux ménagers et domestique, renforcement musculaire modéré, ...) ?

Oui (précisez combien de jours :)
Non

Q19. Au total, combien de temps avez-vous passé à faire des activités modérés au cours des 7 derniers jours ?

Consigne : Précisez le nombre d'heures et minutes sur l'ensemble de la période (par exemple : 10 h 30 sur les 7 derniers jours)

Nombre d'heures : ___
Nombre de minutes : ___

Q20. Pensez au temps que vous avez passé à marcher au cours des 7 derniers jours. Cela comprend la marche sur le lieu de travail et à la maison, les trajets, la marche pour aller faire des courses et tout autre type de marche que vous auriez pu faire pendant votre temps libre pour la détente, le sport ou les loisirs
Au cours des 7 derniers jours, y a-t-il eu de jours au cours desquels vous avez marché pendant au moins 10 minutes d'affilée.

Oui (précisez combien de jours :)
Non

Q21. Au total, combien de temps avez-vous passé à marcher (pendant au moins 10 minutes d'affilée) au cours des 7 derniers jours ?

Consigne : Précisez le nombre d'heures et minutes sur l'ensemble de la période (par exemple : 5 h 30 sur les 7 derniers jours)

Nombre d'heures : ___
Nombre de minutes : ___

Q22. Au cours des 7 derniers jours, combien de temps avez-vous passé assis pendant un jour de semaine en moyenne (hors weekend) ?

Consigne : Précisez le nombre d'heures et minutes sur une journée type

Nombre d'heures par jour : ___
Nombre de minutes par jour : ___

Q23. Combien d’heures passez-vous à dormir vous en moyenne par jour ?

Consigne : Par jour, on doit comprendre "de minuit à minuit" Précisez le nombre d'heures et minutes

Nombre d'heures par nuit : ___
Nombre de minutes par nuit : ___

Q24. Après une nuit habituelle (hors vacances et week-end), comment vous sentez-vous le plus souvent au réveil ?

En forme
Reposé(e)
Un peu fatigué(e)
Très fatigué(e)

Q25. Fumez-vous (du tabac) ?

Non
Oui, occasionnellement
Oui, régulièrement
Je suis en cours d’arrêt / sevrage

Q26. Êtes-vous actuellement enceinte ?

Oui
Non
Je ne suis pas concernée

Message affiché à l'écran : Intéressons-nous maintenant à vos habitudes d'achat ou de consommation alimentaire.

Q27. Dans votre foyer, qui s’occupe le plus souvent de faire les courses alimentaires ?

C’est moi-même
C’est mon conjoint(e)
C’est mon père
C’est ma mère
C’est une autre personne

Q28. Dans votre foyer, qui s’occupe le plus souvent de faire la cuisine ?

C’est moi-même
C’est mon conjoint(e)
C’est mon père
C’est ma mère
C’est une autre personne

Q29. Voici plusieurs questions sur l'utilisation de matière grasse dans votre alimentation. Quelles matières grasses utilisez-vous le plus souvent pour les différents usages proposés ?

Éléments proposés :

Pour la cuisson des viandes à la poêle ou au four
Pour la cuisson des œufs en omelette, au plat
Au moment de servir la viande
Pour la cuisson du poisson à la poêle ou au four
Au moment de servir le poisson
Pour la cuisson des légumes à la poêle
Pour la cuisson du riz ou des pâtes
Au moment de servir les légumes
Au moment de servir les féculents (riz, pâtes,…)
Pour la cuisson des frites
Pour l'assaisonnement des crudités

Modalités de réponse (pour chaque élément) :

Je n'utilise pas de matière grasse pour cet usage / je ne consomme pas cette préparation
01. beurre doux non allégé
02. beurre salé non allégé
03. beurre allégé
04. margarine ordinaire, 70% ou 80% matière grasse
05. margarine au tournesol, 70% ou 80% matière grasse
06. margarine ordinaire, 60% matière grasse
07. margarine à l'huile d'olive, 60% matière grasse
08. margarine au tournesol, 60% matière grasse
09. margarine type "primevère", 60% matière grasse
10. margarine allégée, 40% matière grasse
11. margarine allégée demi-sel, 40% matière grasse
12. margarine "Pro-activ" de Fruit d'or
13. végétaline
14. crème fraîche non allégée
15. crème fraîche allégée
16. huile d'arachide
17. huile mélangée (type Isio 4)
18. huile de tournesol
19. huile d'olive
20. huile de colza
21. huile de maïs
22. autre huile
23. gras de bœuf, saindoux ou autre graisse animale
24. mayonnaise
25. vinaigrette

26. vinaigrette allégée
27. autre, précisez

Q30. Vous arrive-t-il de recevoir des gens à déjeuner ou à dîner, chez vous ?

Oui, régulièrement
Oui, occasionnellement
Non

Q31. Au cours des 12 derniers mois, dans votre foyer, avez-vous déjà réduit les portions de vos plats ou sauté des repas parce que vous n’aviez pas assez d’argent pour la nourriture ?

Oui
Non

Q32. À quelle fréquence cela est-il arrivé ?

Presque tous les mois
Certains mois mais pas tous les mois
Seulement 1 ou deux fois sur l’année

Q33. Au cours des 12 derniers mois, avez-vous personnellement, déjà mangé moins que vous auriez dû, selon vous, parce qu’il n’y avait pas assez d’argent pour acheter de la nourriture ?

Oui
Non

Q34. Au cours des 12 derniers mois, avez-vous, personnellement, déjà eu faim sans pouvoir manger parce que vous n’aviez pas les moyens financiers d’avoir assez de nourriture ?

Oui
Non

Q35. Dites-moi si les situations suivantes vous sont arrivées au cours des 12 derniers mois ?

Éléments proposés :

La nourriture achetée n'était plus en quantité suffisante, et l'argent manquait pour en acheter plus.
C'est difficile (financièrement) de manger des repas équilibrés.

Modalités de réponse (pour chaque élément) :

C’est arrivé souvent
C’est arrivé parfois
Ce n’est jamais arrivé

Q36. Dans votre entreprise, bénéficiez-vous...?

Consigne : Plusieurs réponses possibles

D’un restaurant d’entreprise
D’un lieu aménagé sur votre lieu de travail (avec une table, un réfrigérateur et/ou un four mis à votre disposition)
De titres de restaurant (Ticket restaurant, Chèques déjeuner, Chèques restaurant, Chèques de table, Carte Chèque déjeuner)
Il n'y a rien de cela

Q37. Quel est le montant de vos titres de restaurant (pour une journée de travail) ?

(Réponse libre) Champ(s) proposé(s) :

__ euros __ centimes

Q38. Utilisez-vous vos tickets essentiellement pour vos déjeuners lorsque vous travaillez ?

Oui
Non

Q39. Dans quels lieux de vente utilisez-vous les tickets restaurants ?

Consigne : Plusieurs réponses possibles

Dans les restaurants
Dans un restaurant d’entreprise
Dans une boulangerie, vente à emporter
Dans un supermarché, épicerie, magasins de fruits et légumes

Q40. Durant les 3 jours d'enquête, avez-vous utilisé un ticket restaurant ?

Consigne : Plusieurs réponses possibles

Oui, le premier jour de remplissage
Oui, le deuxième jour de remplissage
Oui, le troisième jour de remplissage
Non, je n'en ai pas utilisé pendant les 3 jours d'enquête

Q41. Quel est le budget d'alimentation par semaine de votre ménage pour les repas pris à la maison y compris les boissons (hors repas extérieurs) ?

(Réponse libre) Champ(s) proposé(s) :

__ euros

Q42. Êtes-vous préoccupé(e) par les sujets suivants autour de votre alimentation ?

Éléments proposés :

La qualité des produits alimentaires

Le bien-être animal
L’impact environnemental
La juste rémunération des producteurs
La transparence sur l’origine des produits

Modalités de réponse (pour chaque élément) :

Très préoccupé(e)
Plutôt préoccupé(e)
Plutôt pas préoccupé(e)
Pas du tout préoccupé(e)

C- Données anthropométriques et hygiène de vie

Q43. Quelle est votre taille ?

Consigne : Par exemple : 1m70, merci de noter : 1,70

___ m

Q44. Quel est votre poids ?

Consigne : par exemple 70 kg

___ kg

Q45. Faites-vous un régime ?
Avez-vous une alimentation particulière ?

Consigne : Plusieurs réponses possibles

Oui, pour raisons médicales
Oui, je suis végétarien(ne) (c'est-à-dire que je m'abstiens de manger de la viande, du poisson, mais je consomme des œufs, du fromage ou du lait)
Oui, je suis végan ou végétalien(ne) (c'est-à-dire que je m'abstiens de manger de la viande, du poisson, mais aussi tous les produits laitiers et les œufs, et je ne mange que des céréales, des légumes et des fruits)
Oui, j'ai un régime sans lactose
Oui, j'ai un régime sans gluten
Oui, j'ai un régime en raison d’allergies
Oui, pour prendre du poids
Oui, pour maigrir
Oui, pour rester en forme
Oui, pour une autre raison (précisez laquelle)
Non, je ne suis aucun régime particulier

D- Nationalité de l'adulte / Influences culturelles

Message affiché à l'écran : Encore quelques questions vous concernant.

Q46. Êtes-vous ?

D’origine française
Français(e) d’acquisition – a acquis la nationalité française au cours de sa vie
D’origine étrangère
Je ne veux pas répondre

Q47. Quelle est votre nationalité d'origine ?
De quelle nationalité êtes-vous ?

Algérienne
Amérindienne
Angolaise
Belge
Brésilienne
Britannique
Camerounaise
Canadienne
Colombienne
Congolaise
Dominicaine
Égyptienne
Espagnole
Finlandaise
Française
Gabonaise
Haitienne
Indienne
Irlandaise
Italienne
Ivoirienne
Malgache
Marocaine
Mauricienne
Mauritanienne
Polonaise
Portugaise
Roumaine
Sénégalaise
Sri-Lankaise
Suisse
Togolaise
Tunisienne
Turque
Yougoslave

Autre (précisez)
Je ne souhaite pas répondre

Q48. Vous est-il arrivé, au cours de l'année 2023, de manger...?

Consigne : Plusieurs réponses possibles

Des produits halal
Des produits cacher
Ni l'un ni l'autre
Je ne souhaite pas répondre

Q49. À quelle fréquence vous est-il arrivé, au cours de l'année 2023, de manger des produits halal ?

Consigne : Plusieurs réponses possibles

Plus d'une fois par semaine
Une fois par semaine
Deux à trois fois par mois
Une fois par mois
Moins souvent
Je ne sais pas

Q50. À quelle fréquence vous est-il arrivé, au cours de l'année 2023, de manger des produits cacher ?

Consigne : Plusieurs réponses possibles

Plus d'une fois par semaine
Une fois par semaine
Deux à trois fois par mois
Une fois par mois
Moins souvent
Je ne sais pas

Q51. Concernant les religions et la spiritualité, vous diriez-vous ?

Consigne : Plusieurs réponses possibles

Croyant(e) et pratiquant(e)
Croyant(e) mais non pratiquant(e)
Pas intéressé(e) par ces questions

E- Informations sur le foyer (À TOUS)

Message affiché à l'écran : C'est presque terminé ! Encore quelques informations sur votre foyer.

Q52. Vivez-vous dans...?

Une maison individuelle
Un appartement dans un immeuble ou logement collectif

Q53. Votre ménage est-il...?

Locataire
Propriétaire ou en accession à la propriété
Logé gratuitement

Q54. Avez-vous un jardin où vous cultivez des fruits ou légumes ?

Oui
Non

Q55. Parmi les situations suivantes, quelles sont celles qui vous sont déjà arrivées ?

Consigne : Plusieurs réponses possibles

Ne pas avoir de voiture personnelle pour des raisons financières
Ne pas pouvoir maintenir son logement à bonne température pour des raisons financières
Ne pas pouvoir remplacer des meubles hors d'usage pour des raisons financières
Avoir des impayés de mensualités d'emprunts, de loyer ou de factures d'électricité, d'eau ou de gaz
Ne pas pouvoir dépenser une petite somme d'argent pour soi sans avoir à consulter quiconque
Ne pas pouvoir faire face à des dépenses inattendues
Ne pas avoir accès à internet pour des raisons financières
Ne pas pouvoir retrouver des amis ou de la famille au moins une fois par mois pour boire un verre ou pour un repas pour des raisons financières
Ne pas avoir une activité de loisirs régulière pour des raisons financières
Ne pas pouvoir s'offrir une semaine de vacances hors de son domicile pour des raisons financières
Ne pas avoir deux paires de bonnes chaussures pour des raisons financières
Ne pas pouvoir avoir un repas contenant des protéines au moins tous les deux jours pour des raisons financières
Ne pas pouvoir acheter des vêtements neufs
Aucune de ces situations ne m'est arrivée

Q56. Laquelle des affirmations suivantes vous semble décrire le mieux la situation financière actuelle de votre foyer ?

Vous avez pris un (ou plusieurs) crédits pour arriver à boucler votre budget
Vous vivez un peu sur vos réserves
Vous bouclez juste votre budget avec vos revenus
Vous arrivez à mettre un peu d'argent de côté
Vous arrivez à mettre pas mal d'argent de côté

Q57. Pouvez-vous m'indiquer la tranche qui correspond globalement au revenu de votre ménage ?

Consigne : C'est-à-dire vos revenus personnels et éventuellement ceux de votre conjoint(e) (avant impôt et après cotisations sociales, tous revenus confondus : salaires, primes, allocations, pensions, revenus financiers, …)

Moins de 1 000 € / MOIS (ou moins de 12 000 € / AN)
1 000 - 1 199 € / MOIS (ou 12 000 - 14 399 € / AN)
1 200 - 1 399 € / MOIS (ou 14 400 - 16 799 € / AN)
1 400 - 1 699 € / MOIS (ou 16 800 - 20 399 € / AN)
1 700 - 1 999 € / MOIS (ou 20 400 - 23 999 € / AN)
2 000 - 2 299 € / MOIS (ou 24 000 - 27 599 € / AN)
2 300 - 2 999 € / MOIS (ou 27 600 - 35 999 € / AN)
3 000 - 4 499 € / MOIS (ou 36 000 - 53 999 € / AN)
4 500 - 5 999 € / MOIS (ou 54 000 - 71 999 € / AN)
6 000 € ou plus / MOIS (ou 72 000 euros ou plus / AN)
Je ne sais pas
Je ne souhaite pas répondre

Message affiché à l'écran : Merci pour votre participation à l'enquête Nutrimétrie. Vous êtes arrivé(e) à la fin.
La qualité de vos réponses sera examinée dans les prochains jours / semaines. Si elle répond à nos normes de qualité, vous recevrez votre récompense via un e-mail de nutrimetrie@potloc.com, au plus tard le 31 janvier 2025. N'oubliez pas de consultez vos spams.
Si vous avez des questions, veuillez nous envoyer un email à nutrimetrie@potloc.com.
